# Large Language Model–Assisted Annotation Framework for Cross-Platform Analysis of Online Autism Communities: Implications for Parent Education and Digital Support

**DOI:** 10.2196/85290

**Published:** 2026-07-10

**Authors:** Yifan Xu, Jianhao Ma, Yujia Hu, Yixue Liu, Yu Chen, Wei Feng, Changwei Zhang, Lei Zhang, Xuening Zhang, Ruochen Huang

**Affiliations:** 1 Nanjing Medical University Nanjing, Jiangsu China; 2 The Affiliated Wuxi People's Hospital of Nanjing Medical University Wuxi, Jiangsu China; 3 Purple Mountain Laboratories Nanjing, Jiangsu China; 4 National Health Commission Key Laboratory of Contraceptives Vigilance and Fertility Surveillance Nanjing, Jiangsu China; 5 Jiangsu Provincial Medical Key Laboratory of Fertility Protection and Health Technology Assessment Nanjing, Jiangsu China; 6 Jiangsu Health Development Research Center Nanjing, Jiangsu China

**Keywords:** ASD, autism spectrum disorder, health information seeking, large language model, LLM, LLM-assisted annotation, online health community, topic analysis

## Abstract

**Background:**

Online health communities (OHCs) are important channels for families of children with autism spectrum disorder to obtain health information and psychosocial support. Differences between an open forum platform and the physician-patient consultation platforms may shape caregiver decisions, yet comparative evidence from China remains limited. A large language model (LLM) provides a scalable approach for systematic content annotation in large OHC datasets.

**Objective:**

This study proposes and validates a standardized LLM-assisted annotation framework under a unified classification schema and compares topic distributions and poster identities across an open forum platform (Baidu Tieba) and physician-patient consultation platforms (Chunyu Doctor and Haodf).

**Methods:**

We implemented an LLM-assisted annotation framework. A unified taxonomy of topics and poster identities was first developed through human open coding. Poster identities in Baidu Tieba were annotated through a double-blind manual procedure. For topic classification, interannotator and human-LLM agreement were evaluated on a manually labeled subset to benchmark models of varying sizes. The best-performing LLM was selected for full-dataset topic annotation, followed by statistical and cross-platform analysis.

**Results:**

When metrics were arithmetically averaged across all annotation tasks, the best-performing LLM achieved agreement levels comparable to human annotation (accuracy=79.18%, SD 0.20%; κ=0.736, SD 0.003; *F*_1_-score=0.727, SD 0.006), approaching interannotator agreement (accuracy=81.65%; κ=0.767; *F*_1_-score=0.758), demonstrating strong stability and scalability. Full-dataset analysis yielded 3 main findings. First, model performance increased with parameter scale but plateaued beyond 14B, indicating diminishing marginal returns from further scaling. Second, clear cross-platform differences in poster identity were observed: the open forum platform was dominated by family members of patients (caregivers: 2377/3516, 67.61%), with substantial participation from commercial rehabilitation practitioners (commercial posters: 427/3516, 12.14%), resulting in a more heterogeneous participation structure. Third, topic distributions reflected both shared high demand for resource-related information and differentiated help-seeking pathways: both platform types demonstrated consistently high demand for resource recommendation and evaluation; the open forum platform was primarily characterized by diagnosis-related discussions (1183/7535, 15.70%), whereas the physician-patient consultation platforms were centered on intervention-related consultation (2864/7687, 37.26%).

**Conclusions:**

The LLM-assisted annotation framework proposed in this study enables reliable large-scale annotation of OHC data while maintaining high human-LLM agreement and operational stability. Midsized models (eg, 14B) demonstrated favorable cost-performance efficiency. The findings reveal 2 key aspects: the open forum platform exhibits a complex participation structure, and the influence of commercially affiliated actors should not be overlooked; users on both platform types show sustained demand for resource-related information but follow different help-seeking pathways, emphasizing diagnostic exploration and professional intervention, respectively. These results suggest that platform structure and governance mechanisms may shape caregivers’ information access and decision-making. The framework provides a transparent, reproducible, and cost-effective approach for OHC research. All data were deidentified and handled in accordance with relevant platform policies and ethical standards.

## Introduction

### Background and Objectives

Autism spectrum disorder (ASD) is a neurodevelopmental condition characterized by difficulties in social communication and interaction, as well as restricted and repetitive behaviors. According to the Report on the Development of Autism Education and Rehabilitation Industry in China III, more than 10 million people in China are affected by autism-related disorders. Approximately 20% are children, and an estimated 200,000 new cases are reported each year. As the etiology remains unclear and no curative pharmacological treatment is available, ASD management relies primarily on long-term rehabilitation and psychological intervention. Families, therefore, face sustained caregiving responsibilities and decision-making pressure [1-3].

Under conditions of prolonged care and high uncertainty, many parents turn to the internet to obtain health information, emotional support, and practical guidance. Prior studies have shown that, in the management of chronic and developmental disorders, access to autism-related resources and care pathways is often uneven [4], and online channels can help compensate for limited access to offline health care resources [5-7]. Online health communities (OHCs) have thus become important platforms for families affected by ASD to seek information and peer support [8,9]. Their 24-hour accessibility and anonymity lower participation barriers, making support more accessible for families with time constraints or those living in remote areas [10-13].

However, the openness of online environments also introduces challenges related to information quality and commercial influence. Inaccurate or promotion-oriented content may interfere with family decision-making [14-17]. Two main types of OHCs can be distinguished. One consists of open, one-to-many peer forums. The other includes structured, one-to-one physician-patient consultation platforms. The former emphasizes experience sharing and emotional support, whereas the latter focuses on professional diagnostic and treatment consultation. Yet systematic comparisons across different platform structures within the same disease context remain limited.

To address this gap, this study examines 2 representative autism-related platforms in China—an open forum platform (eg, Baidu Tieba) and the physician-patient consultation platforms (eg, Chunyu Doctor and Haodf). Using a unified analytical framework, we aim to compare topic structures, poster identity distributions, and help-seeking pathways across platform types.

### Literature Review and Research Gap

Previous studies have recognized OHCs as important spaces for information exchange, peer support, and decision-making in the management of chronic and developmental conditions, including ASD. However, most research has focused on single platforms, particularly Western social media sites such as Reddit, Facebook, and Twitter [18,19]. Methodologically, studies have largely relied on manual coding or unsupervised topic modeling. In addition, the commercial posters on an open forum platform and their influence on community structure remain underexamined. In addition, user identity in an open forum platform is an important topic [20,21]. Quantitative analysis of identity stratification and participation hierarchies is also limited.

In recent years, large language models (LLMs) have demonstrated strong performance in biomedical and public health text tasks, including topic classification, summarization, and information extraction [22-25]. OHC texts are highly heterogeneous and context-dependent. Under standardized prompts and in-context examples, LLMs can generate candidate labels and improve annotation efficiency and consistency [26,27]. However, the systematic application of LLMs to cross-platform OHC analysis, particularly with agreement-based evaluation of model scale effects and stability, remains limited.

Specifically, existing research remains limited in 3 key respects. First, although LLMs have increasingly been used for text annotation, most existing frameworks still rely on the direct deployment of a prespecified model, with less attention paid to systematic multimodel benchmarking under standardized conditions. Second, research on autism-related OHCs has focused more on topic identification than on comparing poster identity structures across platform types, particularly the visibility and concentration of commercial actors in an open forum platform. Third, although different online health platforms may support different forms of information seeking, fewer studies have examined cross-platform differences in topic distribution and help-seeking pathways under a unified annotation scheme.

To address these gaps, this study conducts a cross-platform comparison of 2 representative autism-related OHCs in China under a unified annotation framework. Specifically, we examine (1) the effectiveness of the annotation framework and the impact of model scale on annotation performance; (2) the distribution of poster identity structures within an open forum platform, with particular attention to the structural positions of commercial actors; and (3) differences in topic distribution and help-seeking pathways across platform types. By systematically analyzing model performance, identity structures, and topic distributions, this study seeks to provide a transparent, reproducible, and cross-platform analytical framework for applying LLMs in OHC research and to inform platform governance and information support strategies.

### Significance and Practical Implications

This study has both methodological and practical significance. From a practical perspective, differences in participation structures and information pathways across OHCs may shape how caregivers access information, evaluate resources, and make decisions; if such differences are not adequately recognized and addressed, the risk of information asymmetry may be further amplified. From a methodological perspective, by systematically examining model scale effects, identity stratification, and differentiated help-seeking pathways, this study provides an empirical basis for understanding patterns of information demand in China’s digital health landscape. The proposed LLM-assisted annotation framework enables transparent, reproducible, and cross-platform analysis of large-scale online community data, thereby offering a methodological foundation for optimizing platform governance, regulating commercial content, and developing more targeted information support strategies.

The remainder of this study is organized as follows. The Methods section describes the data sources, data processing, LLM-assisted annotation framework, and ethical considerations. The Results section presents the evaluation of model scale effects and the cross-platform comparative analysis results. The Discussion section interprets these findings in light of relevant theories and further outlines their practical implications.

## Methods

### Study Design and Framework Overview

**Figure 1:**
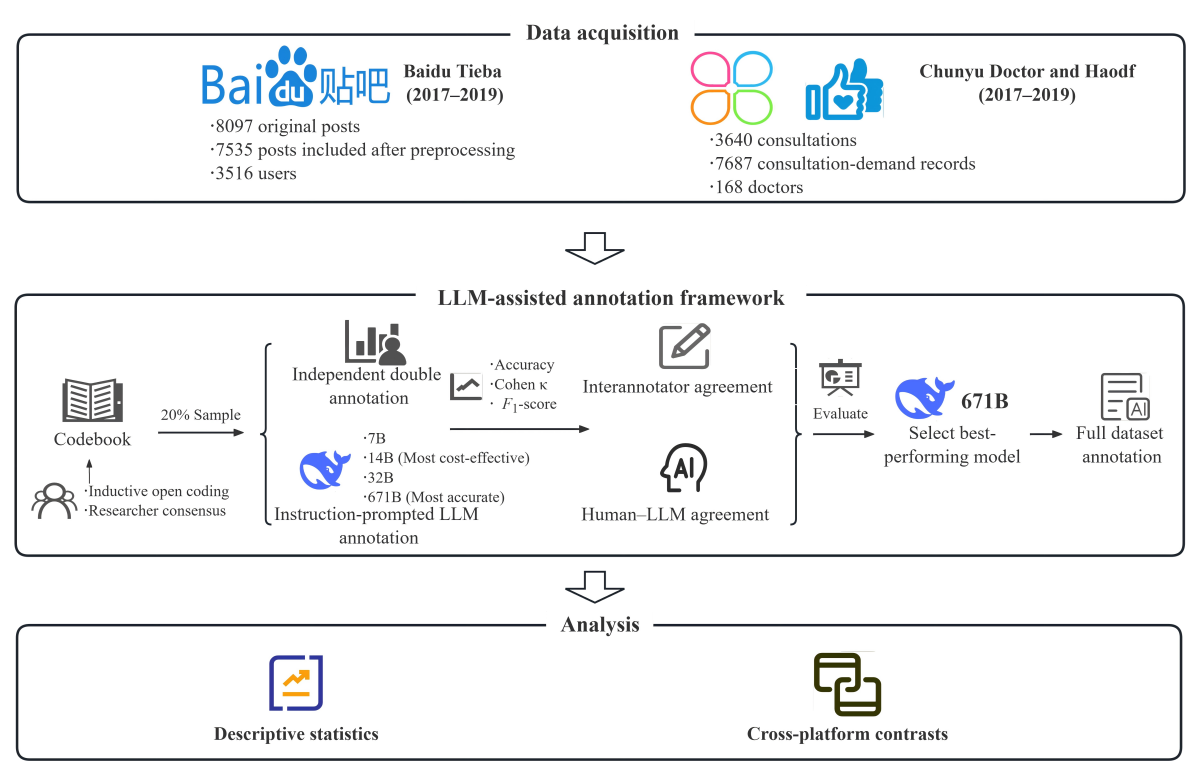
Large language model (LLM)–assisted annotation framework for cross-platform content analysis of autism-related online health communities (OHCs). The framework integrates data collection and preprocessing, human coding scheme development, agreement-based model benchmarking, and full-dataset topic annotation across an open forum dataset from Baidu Tieba and physician-patient consultation datasets from Chunyu Doctor and Haodf.

As illustrated in Figure 1, this study developed, validated, and applied an LLM-assisted annotation framework to conduct a cross-platform comparative analysis of autism-related OHCs.

The methodological content comprised 4 major components: data sources, data processing, the LLM-assisted annotation framework, and ethical considerations.

Through this structured framework, the study enabled scalable, large-scale analysis of heterogeneous community text while ensuring cross-platform comparability, annotation consistency, and methodological transparency.

### Data Sources

This study drew data from 3 representative Chinese OHCs: Baidu Tieba [28], Chunyu Doctor [29], and Haodf [30].

Baidu Tieba is a large topic-based online forum platform in China that follows an open, one-to-many interaction model. Users create themed subforums (“bars”) to initiate posts and engage in discussions. The “Autism” subforum is publicly accessible and includes caregivers, individuals with autism, and related practitioners, forming a typical peer-support communication space.

Chunyu Doctor and Haodf are established internet-based physician-patient consultation platforms in China. They operate under a structured, one-to-one interaction model. Patients or caregivers can consult licensed physicians online to obtain diagnostic advice, intervention guidance, and follow-up communication. These platforms function within regulated service and content governance frameworks.

**Figure 2:**
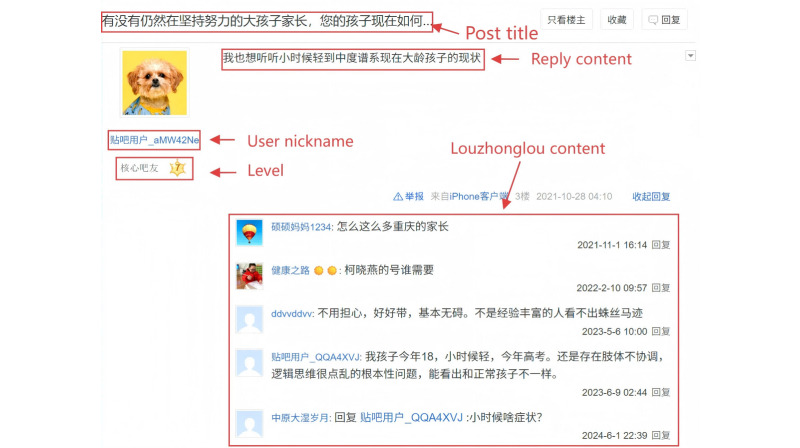
Characteristics of the Baidu Tieba dataset used in this cross-sectional analysis of autism-related online health communities (OHCs) in China, 2017-2019. The figure summarizes the structure of the open forum dataset, including posts, posters, and platform-derived information used for analyses of poster identity, participation level, and topic distribution.

**Figure 3:**
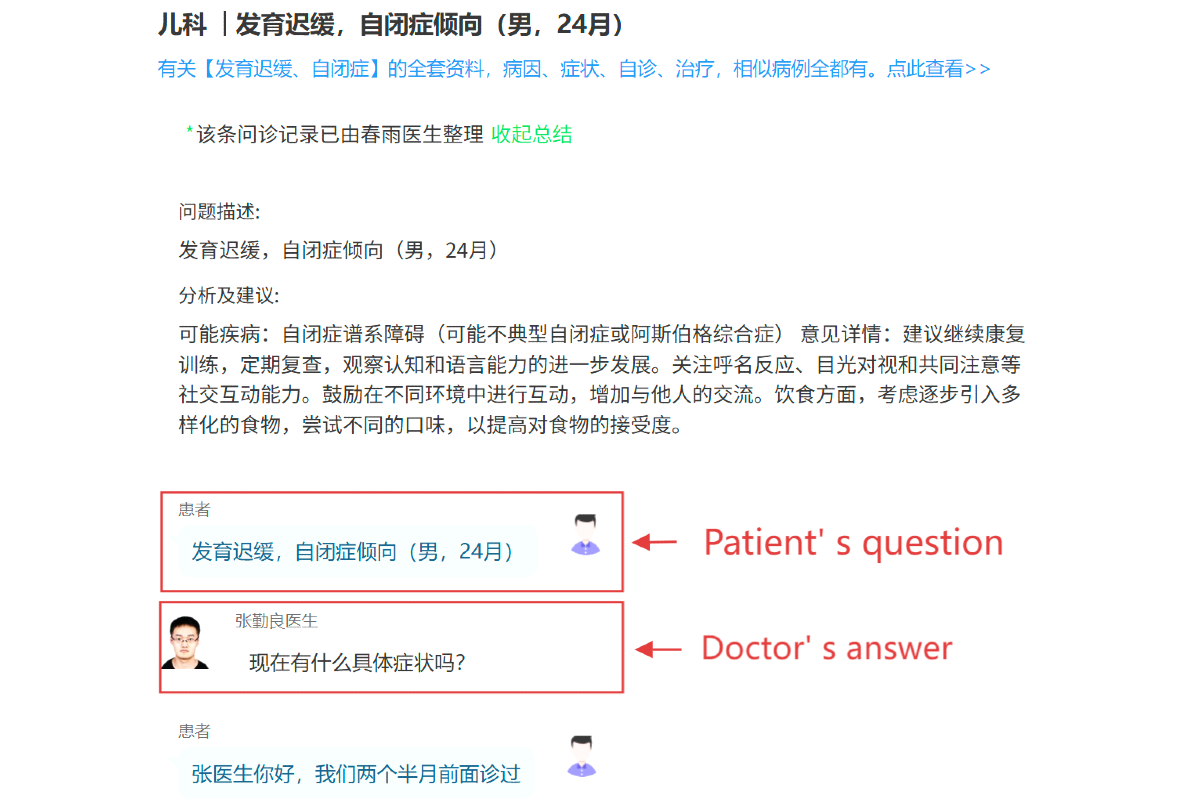
Example of the autism physician-patient consultation record from Chunyu Doctor, a Chinese online consultation platform included in this cross-sectional study of online health communities (OHCs; 2017-2019).

**Figure 4:**
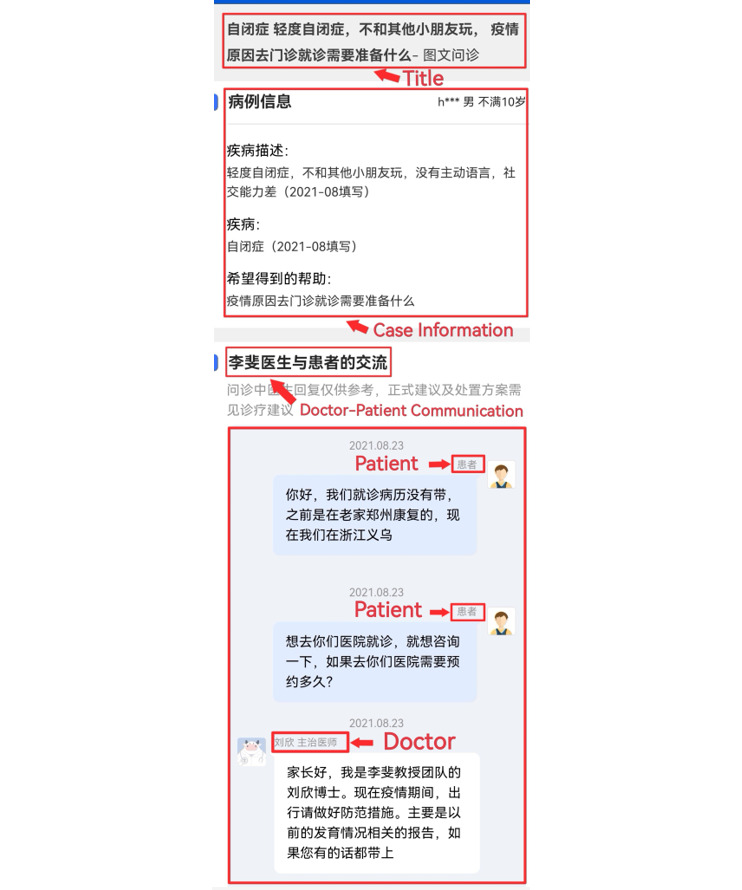
Example of the autism physician-patient consultation record from Haodf, a Chinese online consultation platform, included in this cross-sectional study of online health communities (OHCs; 2017-2019).

Two datasets were assembled as the basis for model validation and substantive analysis: (1) an open forum dataset, derived from the Autism subforum on the Baidu Tieba (hereafter “Baidu Tieba”) open dataset (2017-2019) released by the second “Open Data for Shared Wisdom” National College Open Data Innovation Competition. The dataset included 8097 original posts and 3516 unique posters, representing discussions within an open forum interaction environment (Figure 2); and (2) the physician-patient consultation dataset was collected via Scrapy [31] from publicly available, deidentified consultation records on the Chunyu Doctor (Figure 3) and Haodf (Figure 4) platforms (2017-2019). English translations of the Chinese text shown in Figures 2-4 are provided in Multimedia Appendix 1. The dataset included 3640 physician-patient consultations involving 168 physicians, reflecting help-seeking behavior within structured physician-patient consultation settings.

### Data Processing

Before annotation and analysis, all data were preprocessed to ensure cross-platform data quality and structural consistency. We removed noninformative or abnormal entries (eg, system error strings such as “Http Message object has no attribute getheaders” and empty posts) as well as exact or near-duplicate content. In addition, posts identified as “Unclear Expression” were treated as quality-control exclusion records rather than as a substantive topic category for final analysis. For the open forum dataset, we reconstructed the one-to-one correspondence among thread topics, post content, poster IDs, and poster names to standardize analytical units and ensure traceability of poster-level variables. For the physician-patient consultation platform, core patient consultation content was extracted as the structured unit of analysis to enhance cross-platform comparability. The physician-patient dataset included 7687 consultation-demand records derived from 3640 physician-patient consultations involving 168 physicians. After data cleaning and structural reconstruction, the final dataset included 7535 posts from Baidu Tieba and 7687 consultation-demand records from Chunyu Doctor and Haodf.

### LLM-Assisted Annotation Framework

To enable systematic cross-platform analysis and comparability, we constructed a unified annotation scheme encompassing 3 dimensions: poster identity, discussion topics, and poster participation level.

In this study, interannotator agreement refers to the degree of consistency between the 2 human coders on the labeled subset. Human-LLM agreement refers to the degree of consistency between LLM-generated labels and the human reference labels on the same subset and was used to assess whether model outputs could approximate human annotation reliability. Both types of agreement were evaluated using accuracy, *F*_1_-score, and Cohen κ.

Accuracy was computed as the proportion of correctly classified instances:



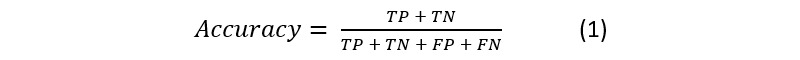



where *TP*, *TN*, *FP*, and FN denote true positives, true negatives, false positives, and false negatives, respectively.

Cohen κ coefficient was used to measure agreement beyond chance:



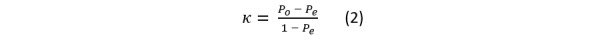



where *P*_*o*_ denotes the observed agreement, and *P*_*e*_ denotes the expected agreement by chance. According to commonly accepted methodological guidelines, κ values between 0.61 and 0.80 are generally interpreted as indicating substantial agreement, whereas values above 0.80 indicate near-perfect agreement.

Precision and recall were defined as:



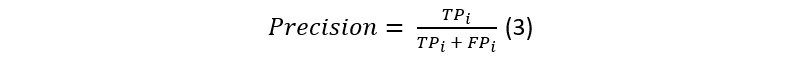





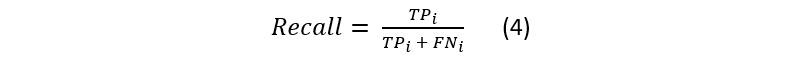



The *F*_1_-score was calculated as the harmonic mean of precision and recall:



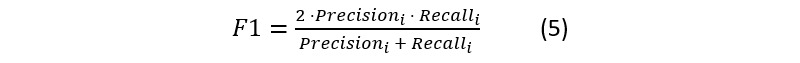



In Baidu Tieba, poster identities were identified and classified using a coding manual (Multimedia Appendix 2). A double-blind manual annotation procedure was conducted to categorize posters into 4 groups: patients, family members of patients (caregivers), commercial rehabilitation practitioners (commercial posters), and others. Two coders independently assigned labels without access to each other’s judgments. After interannotator agreement was evaluated, discrepancies were resolved through discussion to obtain consensus labels for characterizing the composition of different participant groups.

The thematic classification framework was developed based on the Baidu Tieba dataset. Using inductive open coding, the research team established a unified 2-level taxonomy (primary and secondary topics). Primary topics refer to the broad communicative function of a post, whereas secondary topics refer to more specific thematic categories. Because content on a physician-patient consultation platform is inherently help-seeking in nature, all consultation-demand records were classified within the seeking help primary-topic framework, and only the secondary-topic categories were applied. The secondary help-seeking themes were derived from the open coding results. Without altering the overall structure, the taxonomy was adapted for application to the Chunyu Doctor and Haodf consultation dataset, thereby ensuring cross-platform consistency and comparability. Detailed classification rules are provided in the coding manual.

In addition, to capture participation intensity and role differentiation within Baidu Tieba, we constructed a participation-level variable based on platform-displayed poster rank information. According to predefined thresholds (Table 1), posters were classified into 3 mutually exclusive categories: low-activity posters (fans), moderately active posters (members), and highly active core contributors (leaders). This stratification was used to examine differences in topic engagement and help-seeking behaviors across participation levels.

**Table 1:** Mapping between the analytical participation-level categories used in this study and the original user-rank levels displayed on Baidu Tieba.

Aggregation levels and level names in Tieba		Tieba levels
Fans		
	Primary fans	Level 1
	Medium fans	Level 2
	Advanced fans	Level 3
Members		
	Full members	Level 4-5
	Core members	Level 6-7
	Hardcore members	Level 8-9
Leaders		
	Celebrities	Level 10-11
	Popular models	Level 12-13

In terms of model selection, rather than prespecifying a single model for annotation, this study adopted the DeepSeek series of LLMs as the base models. The DeepSeek models demonstrate strong Chinese language understanding and support scalable parameter configurations, making them suitable for domain adaptation and multiconfiguration benchmarking. Under a unified prompting strategy and annotation framework, we conducted benchmark evaluations across models with different parameter sizes to compare their annotation performance under varying conditions. We used standardized Chinese prompt templates with a fixed structure, including role, task, label set, operational definitions, decision and disambiguation rules, auxiliary use of poster identity information, output constraints and format, and input fields (Textbox 1). After this development stage, the prompt templates were fixed within each task and applied consistently across all evaluated models. Performance differences primarily reflect model-related variation under a shared prompting condition rather than iterative prompt engineering during benchmarking. The specific models evaluated are listed in Table 2. All models performed the annotation task in inference mode with a temperature of 0.1. The complete prompts are provided in Multimedia Appendix 3, and the code and related settings are available in a GitHub repository [32] referenced in the Data Availability statement.

**Prompt structure in large language model–assisted annotation.**

**Role**

- Each prompt defines the model’s role as a Chinese text classification assistant operating under predefined rules for the corresponding annotation task

**Task**

- Each prompt specifies the classification objective, the input information to be used, and the requirement to assign one and only one label

**Label set**

- Each prompt provides an explicit set of allowable labels for the corresponding task

**Operational definitions**

- Each label is accompanied by operational definitions to support consistent interpretation and classification

**Decision and disambiguation rules**

- Each prompt includes decision rules, priority rules, or disambiguation rules for adjacent categories and common boundary cases

**Auxiliary use of poster identity information**

- For tasks that include poster identity, the prompts specify how poster_role may be used as auxiliary information without overriding textual evidence

**Output constraints and format**

- Each prompt requires a constrained JSON-only response with predefined field names and allowable label values

**Input fields**

- Each prompt concludes with the task-specific input fields, such as poster_role, primary_topic, title, content, or text

**Table 2:** Characteristics of the DeepSeek large language models benchmarked for topic annotation in this study of autism-related online health communities in China, including model size and release date.

Model name	Model size (billion)	Release date
DeepSeek-R1-Distill-Qwen-7B	7B	January 20, 2025
DeepSeek-R1-Distill-Qwen-14B	14B	January 20, 2025
DeepSeek-R1-Distill-Qwen-32B	32B	January 20, 2025
DeepSeek-V3.2	671B	December 1, 2025

The framework consisted of 3 stages. In the first stage, a human reference dataset was constructed, and interannotator agreement was assessed. Approximately 20% of the data from both platforms were sampled and independently annotated by 2 researchers, who subsequently resolved discrepancies through discussion. The selection of a 20% sampling proportion was informed by prior methodological studies in LLM-assisted qualitative content analysis, which suggest that this proportion provides a balance between reliability assessment and annotation efficiency [27].

In the second stage, model benchmarking and selection were conducted. Multiple model scales were evaluated on the human reference subset using accuracy, *F*_1_-score, and Cohen κ to assess human-LLM agreement. Each model was run 3 times, and stability was assessed using the SD. Stability was quantified as the SD of accuracy, *F*_1_-score, and Cohen κ across 3 independent runs; lower SD indicates higher stability. Based on agreement performance, stability across repeated runs, and practical deployment considerations, we compared the relative trade-offs among model scales. For full-dataset annotation in this study, we selected the model with the strongest overall agreement and stability in order to minimize error propagation in the final analytical dataset.

To further characterize model errors, we reviewed records in the sampled subset that were misclassified in at least 2 of the 3 repeated runs and defined them as recurrent errors. On this basis, we focused on the recurrent errors exhibited by the final model selected for full-dataset annotation and further examined the distribution of error types accounting for more than 10% across different platform types and classification tasks.

In the third stage, full-dataset annotation was performed. After verifying that the human-LLM agreement reached an acceptable level, the selected model was applied to annotate the entire dataset. The resulting annotations served as the basis for subsequent statistical analysis and cross-platform comparisons.

### Ethical Considerations

The data analyzed in this study included only publicly accessible and anonymized content from online platforms, specifically discussion threads, replies, and question-answer records from Baidu Tieba, Chunyu Doctor, and Haodf. Importantly, no personally identifying information was collected beyond poster aliases, ensuring that the privacy of individuals remained fully protected throughout the study.

To prioritize poster privacy, the research team took deliberate steps to avoid publishing or sharing any raw poster posts or consultation texts in their original form. Instead, the findings presented in this study are derived solely from aggregated and anonymized data. This approach guarantees that no individual poster can be identified from the results, further safeguarding privacy.

Since the research relied exclusively on publicly available and deidentified data, a formal ethics review was deemed unnecessary. However, the research team remained committed to responsible data practices and ensured that all analyses were conducted in compliance with the platforms’ terms of service and applicable data use policies.

## Results

### Evaluation of Proposed Framework

#### Quantitative Agreement Evaluation

Detailed agreement metrics across platform types and classification levels are presented in Tables 3 and 4.

In an open forum platform, interannotator agreement was first assessed for poster identity classification. Identity labeling showed a high level of agreement (accuracy=93.76%; κ=0.876; *F*_1_-score=0.890), indicating that the predefined coding manual provided clear operational criteria for distinguishing among patients, family members of patients, commercial rehabilitation practitioners, and others.

For topic classification, interannotator agreement was also examined at both the primary and secondary topics. For primary topics, interannotator agreement reached 78.31% accuracy (κ=0.693), while secondary topics showed even higher agreement (81.92% accuracy; κ=0.796).

Across model configurations, human-LLM agreement improved with model scale. For the primary topic, the 32B and 671B models achieved the highest agreement levels, approaching human performance. For secondary topics, the 671B model achieved the best overall performance (accuracy=81.53%; κ=0.792; *F*_1_-score=0.749), closely approximating interannotator agreement.

In the physician-patient consultation platform, interannotator agreement was higher overall (accuracy=84.72%; κ=0.811; *F*_1_-score=0.840). Human-LLM agreement followed a similar scaling pattern, with the 671B configuration achieving the strongest results (accuracy=77.85%; κ=0.724; *F*_1_-score=0.759). Smaller models demonstrated substantially lower agreement, particularly the 7B.

**Table 3:** Interannotator agreement and human-LLM^a^ agreement for topic annotation in the open forum platform.^b^

Topic and model			Accuracy (%), mean (SD)		κ, mean (SD)		*F*_1_-score, mean (SD)
Human-LLM agreement on primary topic							
	DeepSeek-R1-Distill-Qwen-7B	73.47 (0.46)		0.619 (0.005)		0.618 (0.010)	
	DeepSeek-R1-Distill-Qwen-14B	75.61 (0.73)		0.652 (0.010)		0.642 (0.004)	
	DeepSeek-R1-Distill-Qwen-32B	78.74 (0.54)		0.697 (0.008)		0.670 (0.008)	
	DeepSeek-V3.2	78.16 (0.13)		0.691 (0.002)		0.672 (0.001)	
Human-LLM agreement on secondary topic							
	DeepSeek-R1-Distill-Qwen-7B	71.47 (0.57)		0.683 (0.006)		0.642 (0.020)	
	DeepSeek-R1-Distill-Qwen-14B	78.22 (0.63)		0.755 (0.007)		0.689 (0.013)	
	DeepSeek-R1-Distill-Qwen-32B	79.21 (0.50)		0.767 (0.005)		0.723 (0.016)	
	DeepSeek-V3.2	81.53 (0.36)		0.792 (0.004)		0.749 (0.015)	

^a^LLM: large language model.

^b^Human interannotator agreement for primary topic labels yielded accuracy=78.31%, κ=0.693, and *F*_1_-score=0.670, whereas agreement for secondary topic labels yielded accuracy=81.92%, κ=0.796, and *F*_1_-score=0.764. The table shows model-human agreement for primary-topic and secondary-topic classification across benchmarked DeepSeek models, evaluated using accuracy, Cohen κ, and *F*_1_-score.

**Table 4:** Interannotator agreement and human-LLM^a^ agreement for topic annotation in the physician-patient consultation platform.^b^

Model	Accuracy (%), mean (SD)	κ, mean (SD)	*F*_1_-score, mean (SD)
DeepSeek-R1-Distill-Qwen-7B	50.46 (0.91)	0.417 (0.011)	0.442 (0.007)
DeepSeek-R1-Distill-Qwen-14B	74.66 (0.16)	0.689 (0.002)	0.698 (0.008)
DeepSeek-R1-Distill-Qwen-32B	76.61 (0.59)	0.711 (0.007)	0.719 (0.002)
DeepSeek-V3.2	77.85 (0.12)	0.724 (0.002)	0.759 (0.003)

^a^LLM: large language model.

^b^Human interannotator agreement yielded accuracy=84.72%, κ=0.811, and *F*_1_-score=0.840. The table shows model-human agreement across benchmarked DeepSeek models, evaluated using accuracy, Cohen κ, and *F*_1_-score.

Notably, performance variation across 3 independent runs was small for all models. The mean accuracy SD across benchmarked conditions was below 0.5%, although several individual conditions exceeded this value. The maximum SD was 0.91% for accuracy, 0.011 for Cohen κ, and 0.020 for *F*_1_-score. Human-LLM agreement levels were comparable to interannotator agreement across classification levels.

The 14B achieved strong agreement performance across platform types. Further scaling to 32B and 671B resulted in incremental improvements in accuracy and agreement metrics. Considering overall agreement, accuracy, and stability across runs, the 671B demonstrated the strongest performance. Although the gains beyond 14B were incremental rather than dramatic, computational constraints were manageable in this study, and the 671B model still provided the best overall performance across platform types and classification levels. It was therefore retained for full-dataset annotation to maximize annotation quality in the final analytical dataset.

#### Qualitative Recurrent Error Analysis

The final selected 671B model produced 887 recurrent errors across all classification tasks. These included 327 recurrent errors in the open forum platform primary-topic task, 213 in the open forum platform secondary-topic task, and 347 in the physician-patient consultation platform task. Table 5 reports only the error types accounting for more than 10% of recurrent errors within each task.

In the open forum platform, at the primary-topic level, the most frequent recurrent errors were “seeking help” being misclassified as “sharing” (89/327, 27.22%), “sharing” being misclassified as “advertisement” (81/327, 24.77%), and “others” being misclassified as “sharing” (56/327, 17.13%). At the primary-topic level, help-seeking in the open forum platform was often embedded in narrative discourse, with posters describing their experiences at length while expressing help-seeking intentions only intermittently. This narrative structure may have increased the likelihood of confusion between “seeking help” and “sharing.”

**Table 5:** Recurrent error types accounting for more than 10% of task-specific recurrent errors across platform types and classification levels (671B model). Only error types accounting for more than 10% of recurrent errors within each task are shown.

Category and gold label			Predicted label		Within recurrent errors, n/N (%)
Recurrent primary-topic errors in an open forum platform					
	Seeking help	Sharing		89/327 (27.22)	
	Sharing	Advertisement		81/327 (24.77)	
	Others	Sharing		56/327 (17.13)	
Recurrent secondary-topic errors in an open forum platform					
	Autism symptom	Autism diagnosis		86/213 (40.38)	
	Autism symptom	Autism intervention		30/213 (14.08)	
Recurrent errors in the physician-patient consultation platforms					
	Autism intervention	Other help-seeking		54/347 (15.56)	
	Autism symptom	Autism diagnosis		52/347 (14.99)	
	Other help-seeking	Autism resource recommendation (evaluation)		38/347 (10.95)	

In the physician-patient consultation platform, the most frequent recurrent errors were “autism intervention” being misclassified as “other help-seeking” (54/347, 15.56%), “autism symptom” being misclassified as “autism diagnosis” (52/347, 14.99%), and “other help-seeking” being misclassified as “autism resource recommendation” (evaluation) (38/347, 10.95%). Compared with the secondary-topic task in an open forum platform, recurrent errors in the physician-patient consultation platforms were more evenly distributed across categories, and the related texts were generally shorter. This suggests that classification difficulty in this setting may be related to the brevity of consultation expressions and the limited contextual information available, while more general expressions of help-seeking were also more likely to be classified as “other help-seeking.”

### Analysis of the Open Forum Platform

#### Poster Identity and Level Distribution

The distribution of poster identities on the open forum platform is presented in Table 6. Among 3516 posters in the open forum platform, caregivers constituted the largest group (2377/3516, 67.61%), followed by posters categorized as others (614/3516, 17.46%) and commercial posters (427/3516, 12.14%). Patients accounted for a relatively small proportion (98/3516, 2.79%), indicating a predominantly caregiver-centered participation structure and limited direct patient representation.

**Table 6:** The frequency and proportion of poster identity categories in an open forum platform (N=3516).

Identity	Frequency, n (%)	Comments
Family members of patients	2377 (67.61)	Family members of individuals with autism participating in community discussions, such as parents or other relatives
Others	614 (17.46)	Individuals or groups that cannot be clearly classified, including students, researchers, or general posters, without explicit role identification
Commercial rehabilitation practitioners	427 (12.14)	Individuals affiliated with or representing commercial rehabilitation services
Patients	98 (2.79)	Self-identified individuals with autism participating in community discussions

To further examine structural differences in participation intensity, posters were categorized into 3 levels based on platform-level activity indicators: fans (low activity), members (moderate activity), and leaders (high activity).

At the fans level, caregivers dominated participation (1244/2005, 62.04%), followed by others (468/2005, 23.34%), commercial posters (217/2005, 10.82%), and patients (76/2005, 3.79%).

At the members level, the proportion of caregivers increased to 75.85% (1109/1462), followed by commercial posters (197/1462, 13.47%), others (137/1462, 9.37%), and patients (19/1462, 1.30%).

At the leaders level, the participation structure became more differentiated. Although caregivers remained the largest group (24/49, 48.98%), the relative proportion of commercial posters increased to 26.53% (13/49), followed by others (9/49, 18.37%) and patients (3/49, 6.12%).

Identity distribution across activity levels exhibited a stratified pattern: caregivers consistently constituted the majority in absolute terms, whereas the relative presence of commercial posters increased among highly active posters.

#### Topic Distribution

The distribution of topics on an open forum platform is presented in Table 7. At the content level, “seeking help” was the most prevalent primary topic (3084/7535, 40.93%), followed by “sharing” (2717/7535, 36.06%) and “advertisement” (1678/7535, 22.27%).

**Table 7:** The frequency and proportion of post topic classification in an open forum platform (N=7535).

Primary and secondary topics			Frequency, n (%)
Seeking help			3084 (40.93)
	Autism diagnosis	1183 (15.70)	
	Autism resource recommendation (evaluation)	758 (10.06)	
	Autism intervention	572 (7.59)	
	Other help-seeking	256 (3.40)	
	Autism symptom	173 (2.30)	
	Etiology and trigger-related inquiry	76 (1.01)	
	Autism examination	52 (0.69)	
	Cost and financial burden inquiry	14 (0.19)	
Sharing			2717 (36.06)
	Case sharing	1666 (22.11)	
	Science popularization	1051 (13.95)	
Advertisement			1678 (22.27)
Others			56 (0.74)

Within the “seeking help” category, the most frequent secondary topics were “autism diagnosis” (1183/7535, 15.70%), “autism resource recommendation (evaluation)” (758/7535, 10.06%), and “autism intervention” (572/7535, 7.59%). Within the “sharing” category, “case sharing” (1666/7535, 22.11%) and “science popularization” (1051/7535, 13.95%) were dominant subtopics.

**Figure 5:**
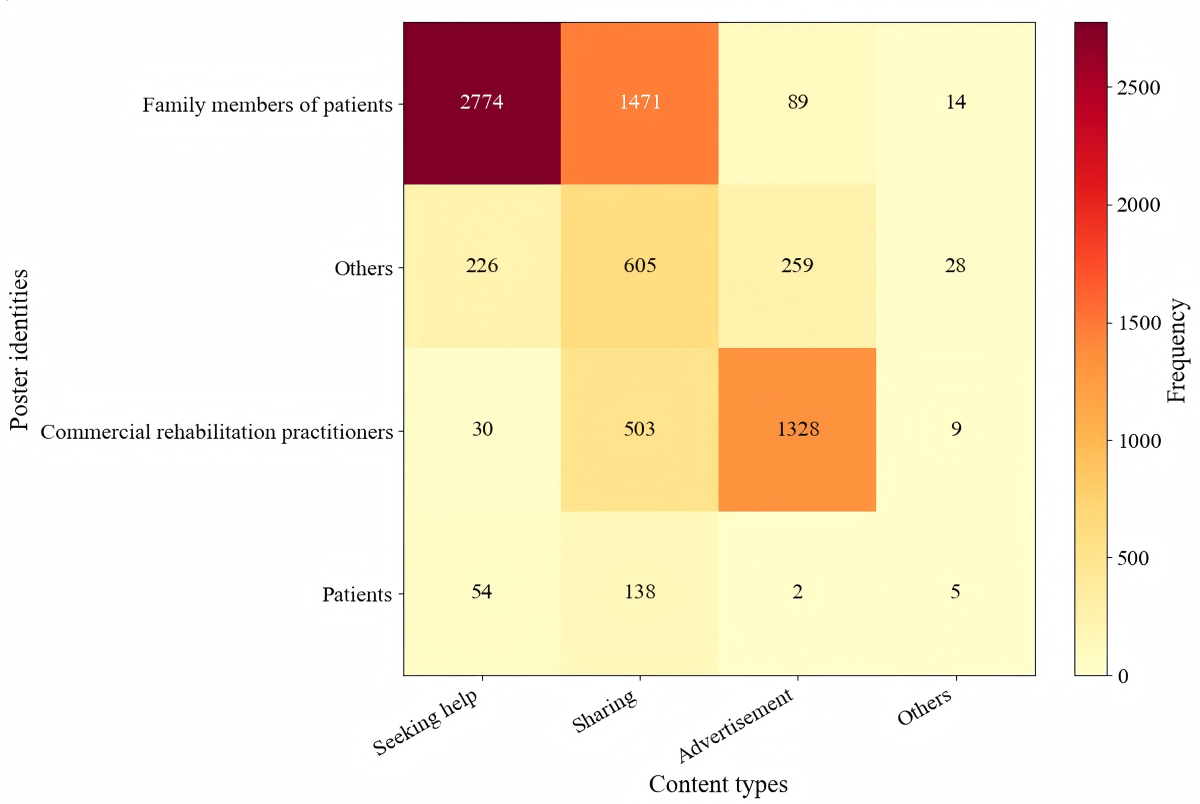
Heatmap of topic distribution across poster identity groups in open forum platform (N=7535 posts). The heatmap illustrates how primary post types were distributed across patients, family members of patients, commercial rehabilitation practitioners, and other posters in the open forum environment.

The identity-topic heatmap (Figure 5) revealed distinct participation patterns at the primary topic. Caregivers were the primary contributors to both “seeking help” and “sharing” posts. In contrast, commercial posters were disproportionately represented in “advertisement” posts, while posters categorized as “others” were more evenly distributed but limited in overall volume.

**Figure 6:**
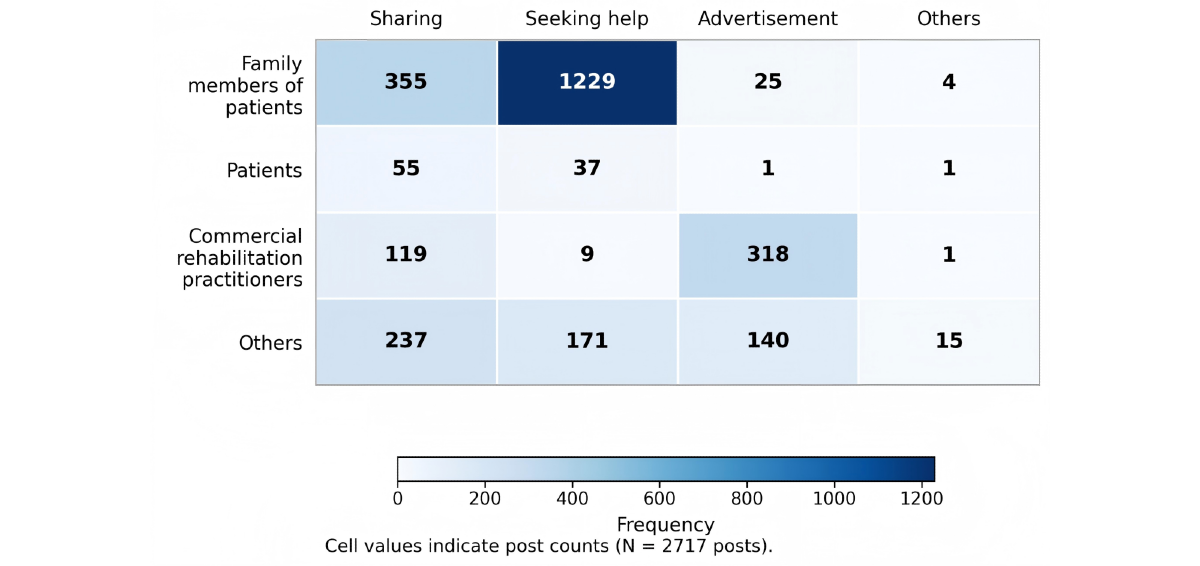
Heatmap of post-type frequency by poster identity among low-activity users at the fans level in an open forum platform. Cell values indicate post counts across poster identity groups and post types (N=2717 posts).

**Figure 7:**
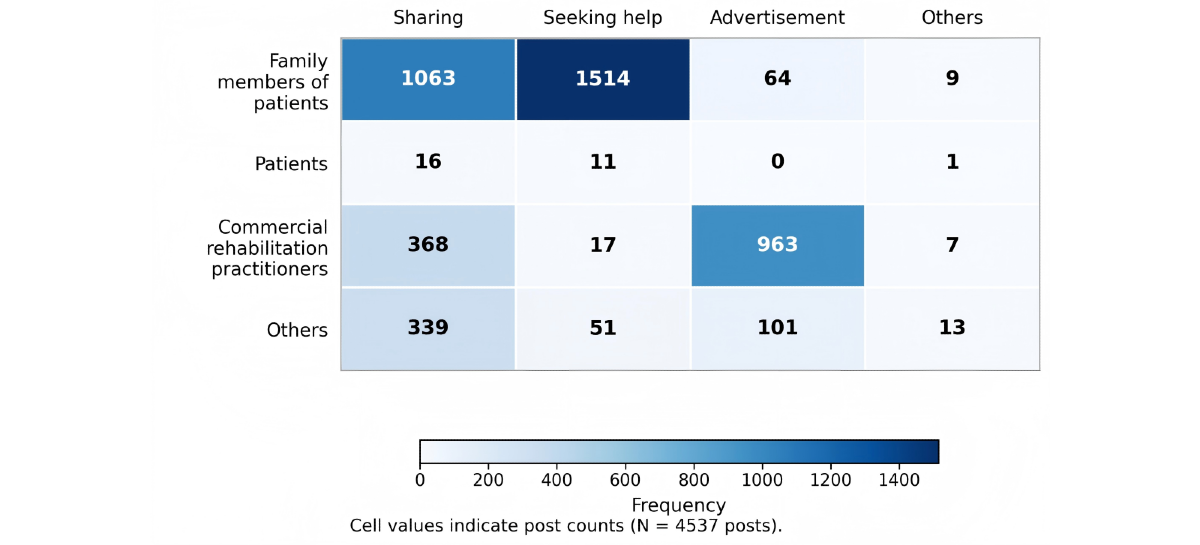
Heatmap of post-type frequency by poster identity among moderately active users at the members level in an open forum platform. Cell values indicate post counts across poster identity groups and post types (N=4537 posts).

**Figure 8:**
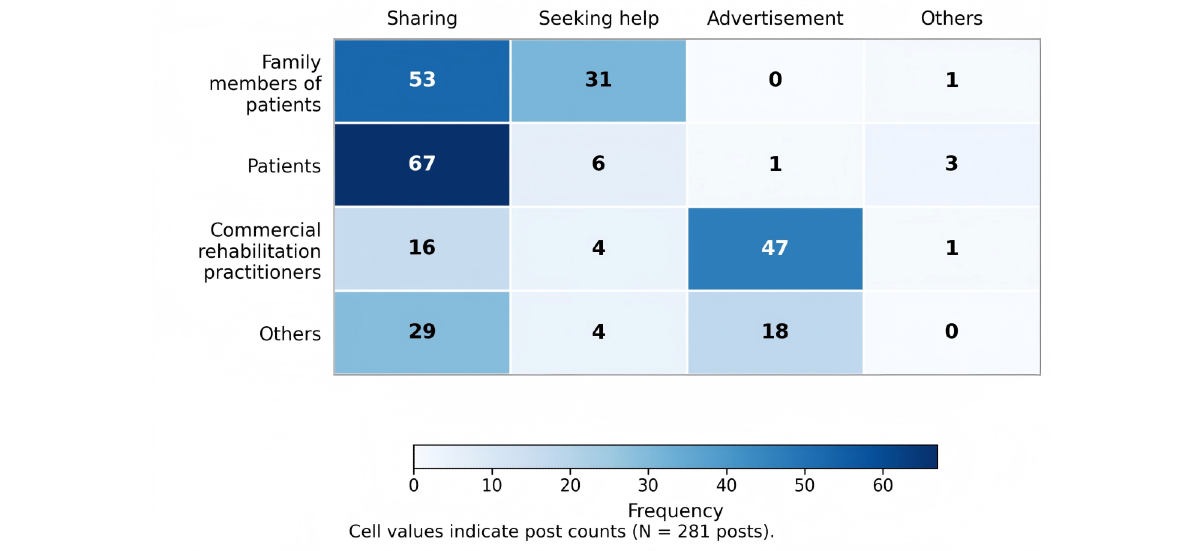
Heatmap of post-type frequency by poster identity among highly active users at the leaders level in an open forum platform. Cell values indicate post counts across poster identity groups and post types (N=281 posts).

Across participation levels, primary topic distributions also showed structural differences (Figures 6-8).

At the fans level, caregivers’ posts were primarily categorized as “seeking help” (1229/1613, 76.19%), followed by “sharing” (355/1613, 22.01%), whereas commercial posters’ posts were predominantly “advertisement” (318/447, 71.14%).

At the members level, caregivers’ posts were distributed between “seeking help” (1514/2650, 57.13%) and “sharing” (1063/2650, 40.11%), while commercial posters continued to post primarily “advertisement” content (963/1355, 71.07%).

At the leaders level, caregivers’ posting patterns shifted toward “sharing” (53/85, 62.35%), exceeding “seeking help” (31/85, 36.47%). Patients also predominantly posted “sharing” content (67/77, 87.01%). Commercial posters remained primarily focused on “advertisement” posts (47/68, 69.12%).

As participation intensity increased, caregivers’ and patients’ posts shifted from “seeking help” toward “sharing,” whereas commercial posters consistently concentrated on “advertisement” across all levels, maintaining relatively high proportions in moderate and high-activity levels.

### Analysis of the Physician-Patient Consultation Platform

After preprocessing and full-scale annotation, a total of 7687 consultation-demand records were included from the physician-patient consultation platforms. In contrast to the open forum platform, discussions on these platforms were more concentrated on clinically oriented concerns.

As shown in Table 8, the most prevalent category was “autism intervention” (2864/7687, 37.26%), followed by “autism resource recommendation (evaluation)” (1358/7687, 17.67%), “autism diagnosis” (1304/7687, 16.96%), and “autism examination” (935/7687, 12.16%). Together, these 4 topics accounted for 84.05% (6461/7687) of all consultation-demand records.

Topics related to “autism symptom” accounted for 6.10% (469/7687), while “other help-seeking” represented 5.28% (406/7687). “Etiology and trigger-related inquiry” comprised 3.53% (271/7687), and “cost and financial burden inquiry” accounted for 1.04% (80/7687).

The physician-patient consultation platforms demonstrated a clinically focused topic distribution, with the majority of consultations concentrated in intervention, diagnosis, resource evaluation, and examination-related categories.

**Table 8:** Frequency and proportion of autistic topics in physician-patient consultation platforms (N=7687).

Topic	Frequency, n (%)
Autism intervention	2864 (37.26)
Autism resource recommendation (evaluation)	1358 (17.67)
Autism diagnosis	1304 (16.96)
Autism examination	935 (12.16)
Autism symptom	469 (6.10)
Other help-seeking	406 (5.28)
Etiology and trigger-related inquiry	271 (3.53)
Cost and financial burden inquiry	80 (1.04)

## Discussion

### Principal Results

#### Overview

In this study, we developed an agreement-based LLM-assisted annotation framework to analyze autism-related OHCs across 2 platform types in China. Model performance improved with increasing parameter size but plateaued beyond mid-sized configurations, suggesting diminishing returns relative to computational cost. Using the validated cross-platform framework, we further compared poster identities, participation hierarchies, and topic distributions between an open forum platform and the physician-patient consultation platforms, revealing shared high-frequency medical concerns alongside clear structural differences in identity composition and communication patterns.

Three major findings emerged from our analysis, as discussed in the following sections.

#### Scale Effects in the Proposed Framework

In the classification tasks examined in this study, model performance generally improved with increasing parameter scale. However, once the parameter size exceeded 14B, gains in agreement and accuracy became more incremental rather than dramatic, suggesting that under the current task complexity and annotation framework design, further scaling did not yield proportional performance improvements. This plateauing pattern was particularly evident at the primary-topic level. At the same time, the substantially lower SD values observed in larger models indicate that scaling mainly improved output stability and reduced stochastic variation across repeated runs.

At the same time, the error analysis suggests that simply increasing model size is unlikely to fully resolve classification difficulty under the current annotation framework. In the open forum platform, recurrent errors were more often associated with relatively long texts and narrative complexity, whereas in the physician-patient consultation platforms, they were more evenly distributed across categories and generally occurred in shorter texts. This indicates that further improvement may depend more on refining category definitions and decision rules than on parameter scaling alone. For long-term deployment or resource-constrained settings, midsized models may still offer a more balanced trade-off between performance, stability, and computational cost.

#### Commercial Visibility and Participation Concentration in the Open Forum Platform

The findings indicate that the participation structure in the open forum platform exhibited marked hierarchical stratification. Although caregivers constituted the vast majority of posters (2377/3516, 67.61%), commercial posters also accounted for a notable proportion (427/3516, 12.14%). Further analysis revealed a clear trend of concentration toward the high-activity end: whereas commercial posters represented a relatively small share at the low-activity tier, their proportion rose sharply to 26.53% (13/49) at the highly active leaders level. This distribution pattern suggests that commercial actors were not uniformly distributed across the platform but strategically occupied high-frequency participation positions, thereby becoming a structural constituent of the platform’s information supply.

From the perspective of stage-based health information propagation, such commercial posters may be understood as a distinct category of senders in the originating stage of the open forum’s information flow [33]. Similar to the substantial proportion of commercial promotional content observed on lifestyle-oriented platforms [34], the present findings suggest that commercial voices also constitute a significant component of the information environment in health-related open forum platforms. For caregivers navigating health uncertainty, this highly visible commercial presence may carry potential risks: prior research has indicated that the dissemination of health misinformation is often driven by financial incentives, and vulnerable populations such as patients and caregivers are particularly susceptible to misleading claims [35].

Unlike a physician-patient consultation platform, an open forum platform typically lacks requirements for conflict-of-interest disclosure and professional gatekeeping mechanisms. Consequently, the high visibility of commercial content in these spaces is not incidental but rather reflects an inherent vulnerability in platform governance. When caregivers seek preliminary guidance in open forum platforms, promotional content and peer-generated experiences often become interwoven within the same information stream, complicating their ability to assess source credibility and discern commercial intent.

#### Information Sufficiency and Platform-Specific Help-Seeking

The results indicate that there are both similarities and differences in topic distribution across the 2 types of platforms. The risk information seeking and processing model suggests that when individuals perceive insufficient information under conditions of health uncertainty, they experience an “information sufficiency gap,” which motivates active information seeking [36]. The findings of this study align with this framework. Across both platform types, discussions were concentrated on core medical topics, including autism diagnosis, intervention, and resource evaluation, reflecting persistent information needs among caregivers.

On the open peer forum, “seeking help” accounted for the largest proportion of posts (3084/7535, 40.93%), with diagnosis-related inquiries particularly prominent (1183/7535, 15.70%). This pattern suggests that some caregivers turn to low-threshold community platforms for preliminary clarification and experiential reference before engaging in formal medical consultation. In contrast, the physician-patient consultation platforms were more concentrated on intervention (2864/7687, 37.26%) and clinical management-related issues, indicating a stronger orientation toward professional guidance.

The risk information seeking and processing framework further emphasizes that individuals adjust their information-seeking strategies based on perceived credibility and accessibility of different channels. The open forum platforms, characterized by lower participation barriers and interactive exchange, may function as spaces for early-stage exploration. The physician-patient consultation platform, by contrast, is more likely to be trusted for in-depth medical advice. Thus, differences in topic distributions across platform types reflect not only structural variation in content but also differentiated information pathways shaped by caregivers’ stage-specific needs.

### Comparison With Prior Work

Existing research on autism-related OHCs has primarily focused on Western platforms, particularly Reddit and other social media sites, emphasizing topic analysis and identity construction. In China, related studies have largely concentrated on open forum platforms such as Baidu Tieba. For example, Fong et al [18] analyzed 740,042 Reddit posts spanning the prepandemic to postpandemic periods and used BERTopic to automatically identify core discussion topics, finding that diagnosis management, intervention choices, and daily coping consistently remained high-frequency themes. Similarly, Deng et al [37] applied Latent Dirichlet allocation topic modeling to 12,667 Baidu Tieba posts and likewise identified diagnosis and intervention as central discussion themes. These findings are highly consistent with the results of this study, suggesting that across both English- and Chinese-language platforms, ASD online communities consistently center their discussions on diagnostic decision-making and intervention-related resources.

However, most existing studies focus primarily on topic structure identification, while systematic quantitative analysis of poster identity composition, participation hierarchy, and cross-platform differences remains limited. Skafle et al [38] qualitatively examined how social media shapes identity formation among individuals with autism, but they did not provide structured measurements of the proportional distribution of different identity groups or their content patterns within communities. Likewise, topic modeling approaches based on Latent Dirichlet allocation or BERTopic generally lack the capacity to identify poster roles or underlying interest affiliations.

Building on this foundation, this study extends the analytical scope by systematically identifying and quantifying the identity structure of posters on Baidu Tieba, including patients, caregivers, and commercially affiliated institutions. It further examines differences in topic distribution and participation intensity across identity groups. The results indicate that in the open forum platform, the proportion of commercial posters increases at higher activity levels, and their contributions predominantly consist of advertisements and resource promotion. This extends prior autism-related OHC research from topic identification alone to the joint analysis of topic distribution, identity structure, and participation hierarchy.

In addition, under a unified secondary topic and identity coding framework, this study conducts a cross-platform comparison between the open forum platform and the physician-patient consultation platform. The findings demonstrate that the physician-patient consultation platform is more concentrated in patient and caregiver identities and is more oriented toward professional intervention and management guidance, whereas the open forum platform exhibits greater identity diversity and content stratification. This design makes it possible to compare how different platform types shape help-seeking pathways and information demand patterns within a common analytical framework.

Methodologically, compared with prior studies that have used LLMs for annotation [39-43], this study offers several improvements. First, rather than deploying a single prespecified model, the framework begins with systematic multimodel benchmarking. Using the DeepSeek series as the base models, we evaluated 7B, 14B, 32B, and 671B configurations under the same fixed prompting strategy. This design makes performance differences mainly attributable to model-related variation under shared prompting conditions, while also allowing model scale effects to be observed directly. In particular, the results show that although performance improved with increasing model size, gains in accuracy and agreement became clearly limited beyond mid-sized models, suggesting diminishing marginal returns from further scaling under the current task design. Such explicit assessment of scale effects and cost-performance trade-offs is relatively uncommon in conventional annotation frameworks. Second, the framework is accompanied by full methodological documentation as well as publicly available code and supporting materials, making it easier for other researchers to adopt, test, and extend it in other domains. In this sense, the framework serves not only for topic annotation itself, but also as a transparent and reusable methodological basis for model selection and large-scale cross-platform analysis.

### Practical Implications and Recommendations

#### Application of the Proposed Framework in Classifying Patient Needs

The results of this study indicate that, under the proposed LLM-assisted annotation framework, LLMs can stably identify and structure patient needs in OHCs. Human-LLM agreement levels were comparable to interannotator agreement, suggesting that LLM-assisted annotation is feasible for continuous need identification and topic classification in large-scale community data.

Based on this framework, LLMs can be used to extract caregivers’ core concerns regarding autism diagnosis, intervention, and resource evaluation at scale, and to transform high-frequency questions into structured question-answer pairs, thereby providing a data foundation for disease-specific knowledge base development and information services.

In addition, the framework is transferable to other disease-related communities, enabling systematic identification of patients’ care-seeking and information needs. The resulting annotated corpora may also support fine-tuning of lightweight domain-specific models, allowing better alignment with the linguistic characteristics of autism-related communities in the Chinese context.

#### Governance and Optimization of Open Forum Platforms

The results indicate that, in the open forum platform, high demand for resource evaluation coexists with substantial commercial content. Low-activity posters were primarily engaged in help-seeking, whereas commercial posters accounted for a relatively higher proportion among high-activity posters. This structure may expose less experienced posters to commercial influence under conditions of uncertainty, potentially leading to information asymmetry.

As poster activity increases, some commercial accounts become frequent contributors. Within the networked communication structure, such highly active posters may exert amplification effects in agenda setting and information diffusion, effectively functioning as key opinion nodes. Therefore, platforms may consider strengthening account verification procedures, improving commercial content labeling and disclosure policies, and enhancing transparency of information sources to reduce potential conflicts of interest and misleading risks.

At the same time, physicians and other professionals should be encouraged to participate in community interactions in a standardized manner, for example, through verified credentials, to enhance the supply of authoritative information. Introducing structured professional support while maintaining openness and poster engagement may help optimize the information environment and improve discussion quality.

### Strengths and Limitations

This study conducted a cross-platform comparative analysis of autism online support within the Chinese cultural and health care context, examining both an open forum platform and the physician-patient consultation platform.

Methodologically, we developed and validated an LLM-assisted annotation framework. The framework enabled large-scale automated text analysis while maintaining annotation consistency and semantic stability, as evaluated by accuracy, *F*_1_-score, and Cohen κ. This approach substantially reduced manual coding burden and demonstrated strong stability and scalability, providing a transferable methodological pathway for OHC research.

Using this framework and drawing on relevant theoretical perspectives, we analyzed the identity structure of posters in the open forum platform, with particular attention to the structural position of commercial posters. Commercial posters accounted for a notable proportion and were more prevalent at higher activity levels. Across platform types, both differences and similarities were observed in topic distribution and expressed needs. These patterns reflect sustained demand for resources under conditions of health uncertainty, as well as users’ adaptive information-seeking strategies based on perceived trust and accessibility of different channels.

Several limitations should be noted. First, the data were collected between 2017 and 2019, before the COVID-19 pandemic. OHCs and social media platforms may have evolved substantially since 2019 in terms of platform governance, commercialization patterns, digital health service use, and users’ online help-seeking behaviors. These changes may affect both participation structures and topic distributions. The COVID-19 pandemic may also have influenced users’ help-seeking behaviors, content, and modalities. Therefore, the findings of this study reflect the baseline characteristics of Chinese-language autism-related online communities before the COVID-19 pandemic, and other contexts warrant further investigation. Second, the linguistic training background and potential biases of the DeepSeek model series may have influenced annotation results. Third, full-dataset topic annotation was based on automated model predictions without exhaustive human correction. Therefore, the final annotated dataset likely retains a certain degree of classification error, which may reduce the precision of fine-grained topic classification and cross-platform comparisons.

Future research could incorporate data from multiple time periods to conduct longitudinal comparisons and validate findings using different types of LLMs or domain-specific fine-tuned models to further enhance the accuracy, robustness, and external applicability.

### Conclusions

This study developed and validated an LLM-assisted annotation framework to systematically analyze topic structures and participation patterns across 2 types of autism OHCs in China. When metrics were arithmetically averaged across all annotation tasks, the best-performing model achieved near-human agreement (accuracy=79.18%, SD 0.20%; κ=0.736, SD 0.003; *F*_1_-score=0.727, SD 0.006) and showed stable performance across repeated runs.

Both platform types demonstrated sustained high demand for resource evaluation, indicating strong information-seeking needs under conditions of health uncertainty. The open forum platform was caregiver-dominated, with a growing proportion of commercial posters at higher activity levels. In contrast, the physician-patient consultation platform showed a more concentrated, clinically oriented topic distribution.

More broadly, these findings should be understood as reflecting the prepandemic stage of Chinese digital health communication, providing a useful baseline for future longitudinal comparison rather than a direct account of present-day platform dynamics.

## Appendix

Multimedia Appendix 1 English translations of platform record images used in the manuscript.

Multimedia Appendix 2 Complete coding manual (codebook) developed for classifying autism online community content and poster identities.

Multimedia Appendix 3 Complete prompt templates used for large language model (LLM)–based topic classification in the Baidu Tieba, Chunyu Doctor, and Haodf.

## Abbreviations

ASD: autism spectrum disorder
LLM: large language model
OHC: online health community

## Data Availability

The datasets generated and analyzed during this study are not publicly available due to platform terms of service and privacy protection requirements, but are available from the corresponding author on reasonable request. The Baidu Tieba dataset and the physician-patient consultation dataset from Chunyu Doctor and Haodf were collected from publicly accessible web pages and deidentified before analysis. To support research transparency and reproducibility, the codebook and analysis scripts generated during this study are available in the GitHub repository cited in the reference list [32].
