# Supplementary material for: Large Language Model–Assisted Annotation Framework for Cross-Platform Analysis of Online Autism Communities: Implications for Parent Education and Digital Support
Source: J Med Internet Res. 2026 Jul 10;28:e85290. doi: 10.2196/85290 (PMC13401076; doi:10.2196/85290)
Supplement: Multimedia Appendix 1 [file jmir_v28i1e85290_app1.docx]

**Multimedia Appendix 1: English translations of platform record images**


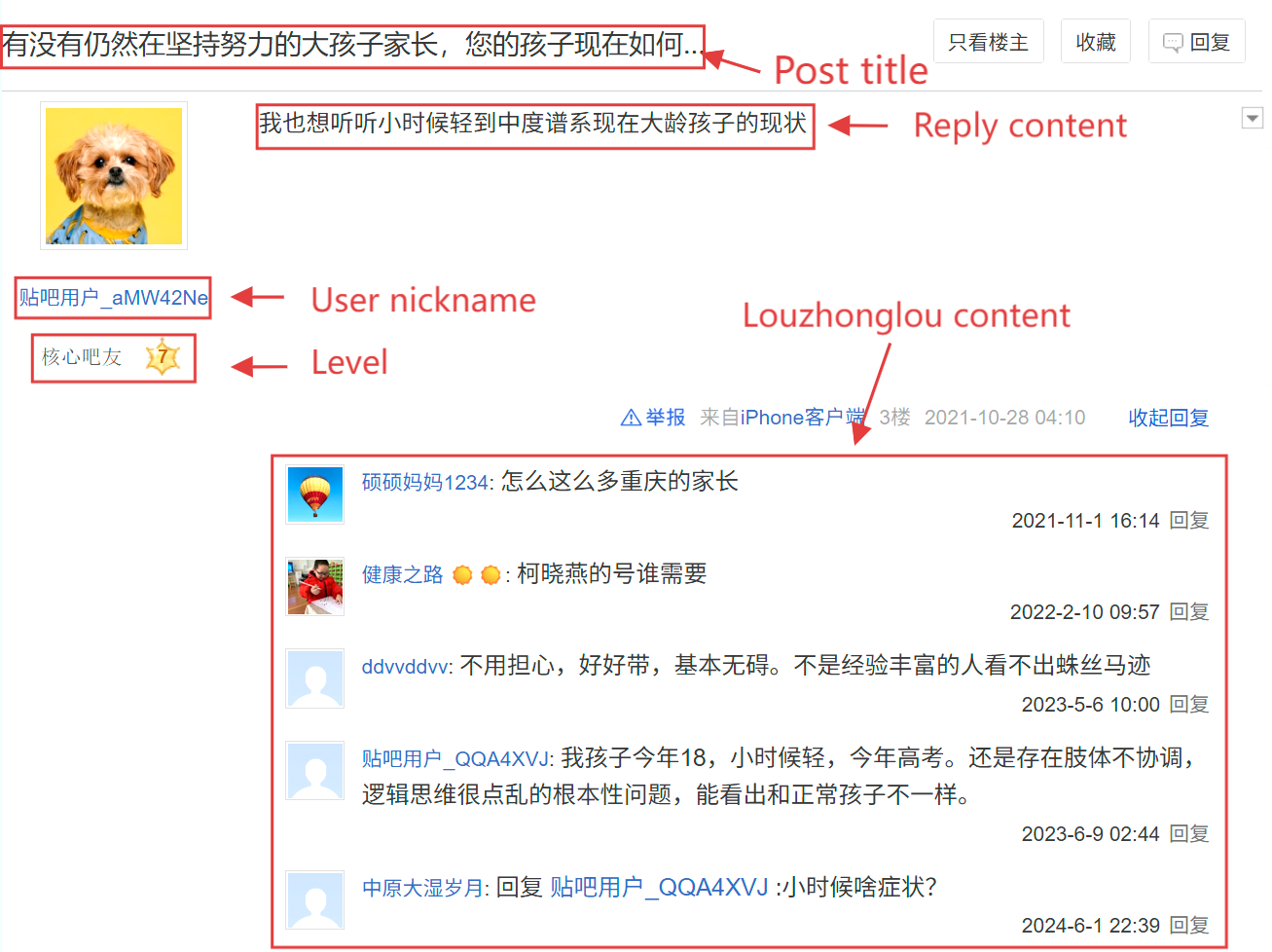


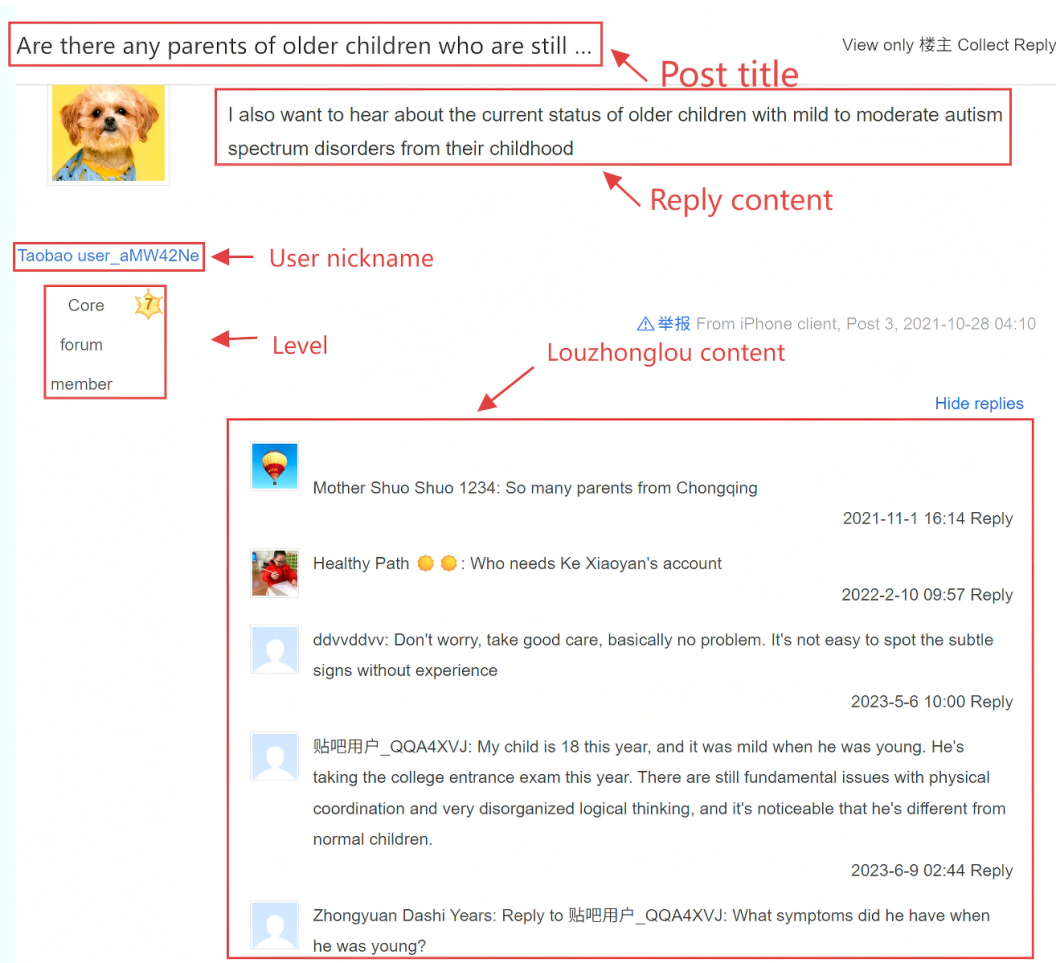


Figure S1. Data characteristics of Baidu Tieba (English translation of original Chinese caption).


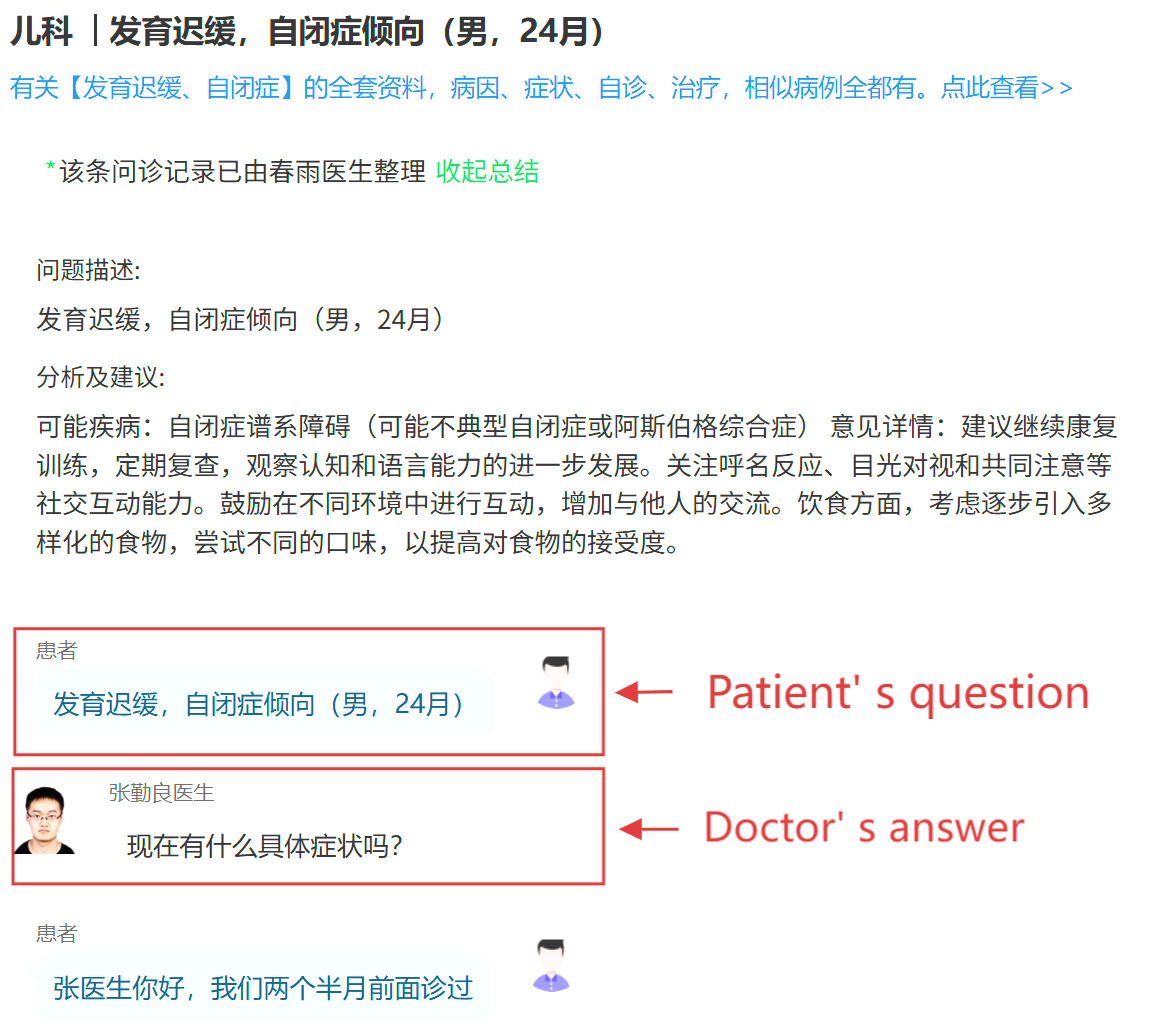


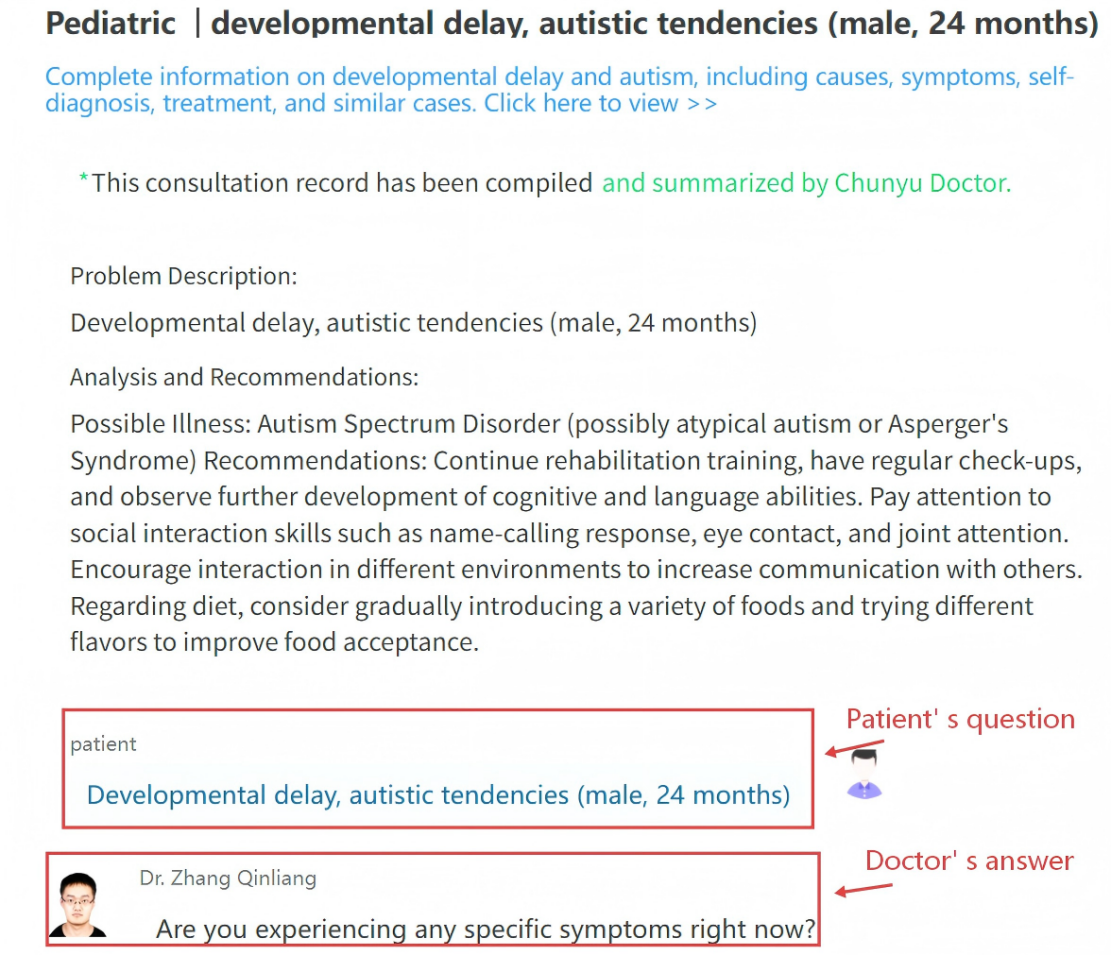


Figure S2. Physician-patient consultation records of Chunyu Doctor (English translation of original Chinese caption).


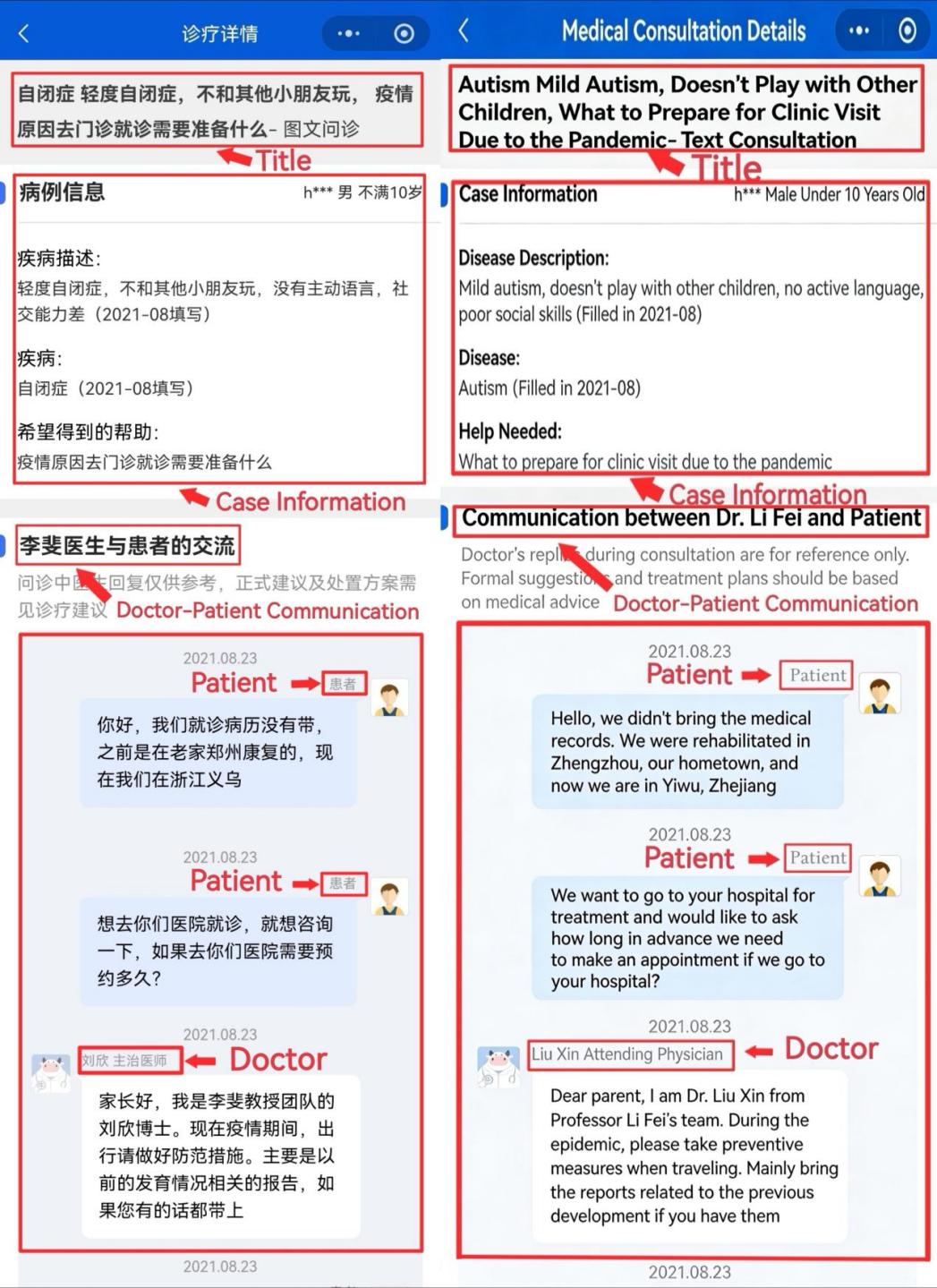


Figure S3. Physician-patient consultation records of Haodf (English translation of original Chinese caption).
