# Supplementary material for: Large Language Model–Assisted Annotation Framework for Cross-Platform Analysis of Online Autism Communities: Implications for Parent Education and Digital Support
Source: J Med Internet Res. 2026 Jul 10;28:e85290. doi: 10.2196/85290 (PMC13401076; doi:10.2196/85290)
Supplement: Multimedia Appendix 2 [file jmir_v28i1e85290_app2.docx]

**Multimedia Appendix 2:** **Coding manual for autism topic and poster identity classification**

**Content**

[1 Purpose and Scope of Classification 3](#_Toc222688227)

[2 Rules for Identity Classification 3](#_Toc222688228)

[2.1 Patients 4](#_Toc222688229)

[2.2 Family members of patients 4](#_Toc222688230)

[2.3 Commercial rehabilitation practitioners 5](#_Toc222688231)

[2.4 Others 5](#_Toc222688232)

[3 Topic Classification Rules for Baidu Tieba 6](#_Toc222688233)

[3.1 Sharing 6](#_Toc222688234)

[3.1.1 Case sharing 6](#_Toc222688235)

[3.1.2 Science popularization 7](#_Toc222688236)

[3.2 Seeking help 8](#_Toc222688237)

[3.2.1 Autism symptom 8](#_Toc222688238)

[3.2.2 Autism examination 9](#_Toc222688239)

[3.2.3 Autism diagnosis 9](#_Toc222688240)

[3.2.4 Etiology and trigger-related inquiry 10](#_Toc222688241)

[3.2.5 Cost and financial burden inquiry 11](#_Toc222688242)

[3.2.6 Autism intervention 11](#_Toc222688243)

[3.2.7 Autism resource recommendation (evaluation) 12](#_Toc222688244)

[3.2.8 Other help-seeking 13](#_Toc222688245)

[3.3 Advertisement 14](#_Toc222688246)

[3.4 Unclear Expression 14](#_Toc222688247)

[3.5 Others 15](#_Toc222688248)

[4 Topic Classification Rules for Chunyu Doctor and Haodf 15](#_Toc222688249)

[4.1 Autism symptom 16](#_Toc222688250)

[4.2 Autism examination 17](#_Toc222688251)

[4.3 Autism diagnosis 17](#_Toc222688252)

[4.4 Etiology and trigger-related inquiry 18](#_Toc222688253)

[4.5 Cost and financial burden inquiry 19](#_Toc222688254)

[4.6 Autism intervention 19](#_Toc222688255)

[4.7 Autism resource recommendation (evaluation) 20](#_Toc222688256)

[4.8 Other help-seeking 21](#_Toc222688257)

# Purpose and Scope of Classification

This codebook is designed to systematically annotate the topics and poster identities of user-generated content in major Chinese online autism communities. It applies to both open forum platforms (e.g., Baidu Tieba) and physician-patient consultation platforms (e.g., Chunyu Doctor and Haodf). The resulting classifications are used for subsequent analyses of topic distribution, cross-platform comparisons, and evaluation of the effectiveness of the large language model (LLM)-assisted annotation framework.

This codebook applies to both human annotation and large language model (DeepSeek)-assisted annotation. On the open forum platform, the unit of classification is a single post, including only the original post text published by the author and excluding any replies. Each classification unit corresponds to one primary topic and one secondary topic. On physician-patient consultation platforms, a single physician-patient interaction may contain multiple distinguishable patient needs. In this study, each distinct patient need is treated as the minimal unit of classification, and each unit corresponds to only one topic.

# Rules for Identity Classification

These rules apply to identity classification on the open forum platform (e.g., Baidu Tieba), based on the poster’ s self-descriptions, linguistic features, keywords contained in the text, and behavioral patterns reflected in their previous posting records.

The identification process is conducted by examining whether all original posts published by the user contain explicit self-identifying strong evidence (e.g., “I am the child’s mother”), whether there are consistent and stable forms of address and narrative patterns supporting a particular identity (e.g., “my child”), and whether there are identity-related keywords, username cues, or other indicative signals.

## Patients

**Definition:** Refers to individuals with autism who post in the first person, explicitly identifying themselves as the patient and sharing their own illness experiences, related experiences, or seeking help. Posts are also classified into this category when the grammatical subject is omitted but the overall context clearly indicates that the author is expressing personal illness experiences or emotions.

**Keywords:** “I,” “myself,” “my own experience(s),” “when I was a child,” “previously,” etc.

**Example records:**

1. “I’ve had autism for more than 20 years! I’m 30 this year!! Still not better and don’t know what to do.”
2. “An autistic college student’s self-narrative: giving some hope to parents whose child has just been diagnosed.”

## Family members of patients

**Definition:** Refers to parents, guardians, or other relatives of individuals with autism who post in the capacity of caregivers. They may explicitly identify themselves as parents/guardians/relatives, or describe the child using expressions such as “my child,” “our baby,” “my son,” “my daughter,” “boy,” or “girl,” while discussing caregiving experiences, medical consultations, or seeking help.

**Keywords:** “my child,” “my kid,” “my daughter/son,” “girl,” “boy,” etc.

**Example records:**

1. “We are from Shanxi. Looking for reliable autism rehabilitation institutions in Beijing.”
2. “May I ask whether my child might have autism?”

## Commercial rehabilitation practitioners

**Definition:** Refers to posters who speak on behalf of rehabilitation institutions, training organizations, or related service providers, including individual business operators or special education practitioners seeking employment. Such posts typically contain clear promotional or advertising intentions and are written from the perspective of an institution or practitioner. Usernames may explicitly indicate institutional affiliation. The purpose of these posts may include attracting traffic, student recruitment, course promotion, paid consultation services, or statements that guide parents toward choosing specific institutions.

**Keywords:** “lecture,” “professor,” “instructor,” “children’s training center,” “professional,” “institution,” “custody service,” “rehabilitation center,” “rehabilitation education,” “training center,” “add me (contact information),” “school for autistic children,” “discount,” “Sino-US Stars,” “welcome to join,” “recruitment,” etc.

**Example records:**

1. “What should you do if a child with autism refuses to attend group classes? Never force them—Shanghai Yuzhixing.”
2. “Enrollment information for full-time care services for special children in Chengdu.”

## Others

**Definition:** Includes user types that do not fall into the above three categories, such as official volunteer group accounts, non-profit organizations, and public accounts without commercial intent. When the text does not contain explicit self-identification, stable identity-related expressions, or other reliable cues supporting identity determination, and no distinctive behavioral features are evident, no inferential classification should be made; instead, the post should be uniformly categorized as “Others.”

**Example records:**

1. “Happy New Year.”
2. “[cp]Happy Mother’s Day to all mothers! Wishing all the little stars a bright future!”

# Topic Classification Rules for Baidu Tieba

Except for posts with missing data, primary and secondary topic classification criteria are established for all remaining posts. Missing data include posts containing crawler error messages (e.g., “'HTTP Message' object has no attribute 'getheaders'”) and reposted or shared posts whose original content has been deleted.

Topic classification is determined based on the core communicative intent and functional content of the post. It is primarily used to identify whether the user is sharing information, seeking help, engaging in commercial promotion, or whether the topic cannot be determined due to insufficient information. In the annotation process, the primary topic should be identified first, followed by the specification of the corresponding secondary topic under that primary category. Given the characteristics of open forum platforms, when a post simultaneously exhibits features of multiple topics, classification should be based on the poster’s primary communicative intent and their identity.

## Sharing

This category includes posts primarily intended to actively share information, knowledge, or experiences. The poster’s main purpose is not to seek help, but to provide content that can be referenced, disseminated, or reused by other community members.

### Case sharing

**Definition:** Refers to posts in which users proactively share personal or family experiences related to autism, including the diagnostic process, intervention experiences, educational experiences, or daily life records, with a clear purpose of experience exchange. Posts that merely state an intention to “talk about the child” without providing substantive content are not included. There is no evident help-seeking intent; rather, the primary purpose is to share stories, exchange experiences, or express personal reflections. This category mainly involves patients or family members of patients and typically describes real experiences or long-term processes, such as initiating a thread to document and update progress over time. The primary purpose is not to ask questions, seek advice, or engage in marketing. Posts that simply state that “the child has autism” without describing specific experiences or processes are excluded from this category.

**Keywords:** “my child,” “our family,” “the child now,” “experience,” “record,” “changes,” “along the way,” etc.

**Example posts:**

1. “My child is 2 years and 8 months old. He doesn’t speak, doesn’t respond when called by name, and rarely plays with other children.”
2. “My child was diagnosed as suspected autism at 2 years and 3 months. Starting this thread to record his situation.”

### Science popularization

**Definition:** Refers to posts in which users proactively share knowledge-based content related to autism, including relevant data, research findings, materials, external links, or images. Such posts have a public education and information dissemination function and do not contain explicit commercial promotion. These posts primarily focus on explanation and description, with a relatively objective and neutral tone. The purpose is to convey information rather than promote specific services or institutions. Sharing information about public welfare activities or educational articles is also categorized as popular science rather than advertising.

**Keywords:** “popular science,” “information sharing,” “research shows,” “key points,” “link attached,” “image explanation,” etc.

**Example posts:**

1. “Autism is not an infectious disease, nor a hereditary disease, nor an organic tissue or organ lesion or gene mutation. Autism is a natural response of biological systems when facing negative thoughts, thinking patterns, logic, understanding, and cognition, accumulating to a certain quantitative level under negative influences. Autism belongs to psychological issues of thought and is classified under the nervous system.”
2. “It is said that autism is due to nutritional deficiency during pregnancy, and it was not supplemented during infancy!”

## Seeking help

This category includes posts primarily intended to actively seek advice, information, experiences, or specific resources from others. Posters are typically in a state of confusion, anxiety, or decision-making, and seek support or guidance through community interaction.

### Autism symptom

**Definition:** Refers to posts in which patients or family members describe specific behavioral manifestations, developmental abnormalities, or daily responses of a child or individual, and seek others’ opinions on whether these behaviors are abnormal or consistent with autism-related characteristics. This also includes questions such as “What are the symptoms of autism?” The core focus of this subcategory is whether the behaviors or manifestations themselves are abnormal, rather than issues related to formal diagnostic procedures or examination results. If the text explicitly asks “Is this autism?” or “How can autism be diagnosed?”, even when a large number of symptoms are described, it should not be classified under this category but instead under the “Autism diagnosis” subcategory. The key criterion for this category is that the discussion centers on behaviors or symptoms themselves, without explicitly inquiring about diagnostic procedures, assessment scales, or specific hospitals.

**Keywords:** “doesn’t speak,” “avoids eye contact,” “doesn’t respond to name,” “stereotyped behaviors,” “unable to communicate,” “strange behavior,” etc.

**Example posts:**

1. “My child is over two years old. He rarely makes eye contact, doesn’t respond much to people, and only plays with toys repetitively by himself. Is there something wrong?”
2. “May I ask if a baby who doesn’t respond when called by name is unusually hyperactive?”

### Autism examination

**Definition:** Refers to posts in which patients or family members raise questions regarding autism-related examination items, assessment tools, or scale results. This includes whether a specific test is necessary, how to interpret examination or scale results, whether completed tests can determine autism, and the role of examinations in the diagnostic process. If examination results are mentioned only as supplementary information and the core question concerns “whether this is autism,” the post should be classified under the “Autism diagnosis” subcategory. The key criterion for this category is that the focus lies on the examination or assessment tool itself, including tests that have been completed or are planned. Even if the ultimate goal is diagnosis, the textual emphasis must be on the testing method or interpretation of results.

**Keywords:** “examination,” “scale,” “ADOS,” “intelligence test,” “assessment form,” “test results,” “whether to do a test,” etc.

**Example posts:**

1. “We’ve already done an intelligence test and an autism scale. What does this score mean? Do we need to do other tests?”
2. “Is it necessary for a child with autism to undergo EEG or MRI?”

### Autism diagnosis

**Definition:** Refers to posts in which patients or family members, based on described symptoms, explicitly focus on determining “whether it is autism.” This includes questions regarding autism subtypes, severity levels, diagnostic likelihood, evaluation conclusions, diagnostic procedures, or confirmation pathways. When a post contains both symptom descriptions and examination results, but the core question centers on “whether this is autism” or “how to obtain a diagnosis (diagnostic pathway),” it should be classified under this category. This includes posts that provide detailed accounts of a child’s behavioral manifestations or developmental history in order to seek a preliminary judgment or advice on diagnostic pathways; posts that present examination or test results as supporting information while asking whether they indicate autism; or posts asking whether additional tests are needed to confirm a diagnosis.

**Keywords:** “Is it autism?”, “please help judge,” “diagnosis,” “suspect,” “let’s discuss,” “I’m very afraid it might be autism,” “what should I do,” etc.

**Example records:**

1. “My child is one year and nine months old! He can’t speak a single word! Very little eye contact! But if he wants to eat something, he will put it in your hand! If you say ‘give me a kiss,’ he will quickly kiss you! When going out, he doesn’t play much with other kids! He likes going up and down stairs! Only plays with same-age children in the family! At home, he likes pouring toys out and putting them back repeatedly! He can’t use a spoon to eat, but can grab biscuits with his hands! Good chewing ability! Loves listening to songs and imitates singers’ movements! The hospital tested trace elements and everything was normal! Please help me see if my child has autism? What other tests should we do?”
2. “Please everyone help me see if my baby has autism! I’m begging you.”

### Etiology and trigger-related inquiry

**Definition:** Refers to posts in which users raise questions regarding the causes, triggering mechanisms, or attribution of responsibility for autism. This includes genetic factors, prenatal or perinatal factors, parenting styles, environmental exposures, and related issues. The core focus of this category is the question “Why did this happen?”—that is, exploring the causes or potential contributing factors of autism. All inquiries specifically concerning the etiology or triggering factors of autism should be classified under this category.

**Keywords:** “genetic,” “did I cause this,” “during pregnancy,” “premature birth,” “vaccine,” “second child,” “family history,” etc.

**Example posts:**

1. “Help: Can cerebral folate deficiency cause autism?”
2. “Is autism related to the formula milk consumed in early childhood?”

### Cost and financial burden inquiry

**Definition:** Refers to posts in which users raise questions about the economic costs and affordability of autism-related diagnosis, treatment, rehabilitation, or long-term interventions. The core focus of this category is the financial cost itself, rather than comparisons of specific institutions. All inquiries regarding expenses associated with autism-related treatment, intervention, or examinations should be classified under this category.

**Keywords:** “cost,” “expensive or not,” “how much was spent,” “reimbursement,” “can we afford it,” etc.

**Example posts:**

1. “How much does one year of rehabilitation cost? Can an ordinary family really afford it?”
2. “My baby has autism. I went to the Disabled Persons’ Federation to apply for subsidies but didn’t get a single cent!! Rehabilitation costs several thousand yuan a month—we simply can’t keep it up.”

### Autism intervention

**Definition:** Refers to posts in which users seek advice regarding professional intervention approaches, treatment pathways, training methods, or implementation strategies for autism, including intervention choices under different subtypes or severity levels, as well as expected prognostic outcomes following intervention. This category specifically concerns professional training or rehabilitation interventions; general guidance in everyday life does not fall under this category. Such posts typically reflect the poster’s concern about intervention outcomes and future development, and essentially relate to decision-making regarding intervention and treatment. However, this category does not include general questions about the impact or severity of autism without reference to specific intervention methods, such as “Is autism serious?” or “Will autism affect the future?” These should be classified according to the specific context under other relevant categories.

**Keywords:** “ABA,” “sensory integration training,” “speech therapy,” “family-based intervention,” “training intensity,” “intervention,” “don’t know what to do,” “rehabilitation methods,” etc.

**Example posts:**

1. “My child is three years and two months old and was diagnosed last week with mild to moderate autism. He can usually make eye contact and look at you when called. He can handle toileting independently and express some simple meanings, but often doesn’t respond to questions and talks to himself. In kindergarten parent-child classes, he doesn’t listen to the teacher and runs around. No stereotyped behaviors. How should this type of autism be rehabilitated? Please advise.”
2. “How can we train a child who avoids eye contact and doesn’t respond when called?”

### Autism resource recommendation (evaluation)

**Definition:** Refers to posts in which users seek recommendations, comparisons, or evaluations of autism-related diagnostic, treatment, or rehabilitation resources. Such resources may include medical institutions (e.g., hospitals or specific departments), individual physicians (including inquiries about which department to consult), as well as rehabilitation institutions or relevant professionals (e.g., rehabilitation teachers, speech therapists, behavior analysts). The core purpose is to select appropriate medical or rehabilitation resources in order to reduce the risk of misdiagnosis or improve intervention outcomes.

**Keywords:** “institution,” “hospital,” “doctor,” “teacher recommendation,” “rehabilitation center,” “reliable or not,” “any pitfalls,” “cost,” etc.

**Example posts:**

1. “Are there any well-regarded rehabilitation institutions in Shenzhen that you would recommend?”
2. “Help! Which hospital in Tianjin can conduct food intolerance testing for children? Not allergy testing.”

### Other help-seeking

**Definition:** This category serves as a residual (catch-all) category and should be used only when a post contains a clear help-seeking intent but cannot be reliably classified into any existing secondary subcategory. During classification, annotators should first attempt to assign the post to the specific subcategories listed above; this category should be used only when no appropriate match can be made. Posts may be classified into this category when they express a clear help-seeking intent but do not meet the criteria of any specific subcategory; involve mixed issues such as autism combined with other medical conditions; contain multiple intertwined questions that are difficult to further disentangle; or address topics not directly related to autism. Additionally, posts with highly generalized or unclear problem descriptions, where the poster’s specific needs are difficult to identify, may also be included in this category.

**Example posts:**

1. “Baby has cow’s milk protein allergy. How is Aminogen? Also a bit lactose intolerant.”
2. “In my previous workplace, I saw a student suddenly have an epileptic seizure, with uncontrollable body movements, several people holding him down like an electric shock. So what are the symptoms of epilepsy in babies?”

## Advertisement

**Definition:** Refers to posts primarily intended to promote products, courses, services, or institutions, including both direct advertisements (hard ads) and indirect advertisements (soft ads) that are framed as popular science or experience sharing. If the poster is a patient or family member who merely shares personal experiences, feelings, or non-profit mutual support information, and does not include traffic diversion, contact information, course details, or pricing information, the post should not be classified as an advertisement. Similarly, parent-initiated group formation for communication about autism, without promoting specific products or services, should not be considered advertising. The core characteristic of this category is that the post aims to attract traffic, recruit participants, or promote courses or services, often accompanied by contact information or calls to action.

**Keywords:** “institution name,” “rehabilitation center,” “registration,” “trial class,” “discount,” “add me,” “WeChat ID,” “phone number,” “QR code,” etc.

**Example posts:**

1. “XX Rehabilitation Center has launched a new ABA course. Scan the QR code to book a trial class. Limited spots available.”
2. “Urgently hiring one to two special education teachers in Bazhou, Langfang, Hebei. Check inside for salary details.”

## Unclear Expression

**Definition:** Refers to posts whose content is incomprehensible or incomplete. Even if keywords appear, they are not directly related to autism, relevant institutions, or the topic at hand (posts containing autism-related terms should not be classified into this category). This category does not include cases of technical data loss (e.g., crawler errors). Such posts typically represent normal posting behavior but contain unclear semantic meaning, inaccessible links, fragmented language, or insufficient substantive content, making it impossible to determine a specific topic or intent.

**Example posts:**

1. “I posted a picture thread. Come and take a look~”
2. “Just saying hi.”

## Others

**Definition:** Refers to posts that cannot be classified into any of the above primary or secondary categories, or that represent rare or marginal situations, such as reports, forum management issues, or other administrative matters. This category also includes posts that express emotional distress or complaints without a clear help-seeking intent, as well as cases where parents form groups for communication about autism or proactively offer support to individuals with autism and their families, without involving specific requests, diagnostic concerns, intervention discussions, or promotional content. Posts in this category are comprehensible in content and do not involve technical data loss. They represent normal posting behavior but have relatively low relevance to the core research themes of this study.

**Example posts:**

1. “Can this be set as a signature now?”
2. “Please care for children with autism.”

# Topic Classification Rules for Chunyu Doctor and Haodf

The annotation of data from Chunyu Doctor and Haodf is adapted across platforms based on the secondary topic system under the primary category of “Seeking help” established for Baidu Tieba. The definitions, keyword scope, and decision logic of the secondary topics remain consistent in principle, and no new topic categories are introduced.

Compared with open forum platform such as Baidu Tieba, Chunyu Doctor and Haodf are structured medical consultation platforms. Their textual content is typically composed of continuous follow-up questions from physicians and segmented responses from patients. Patients’ consultation needs are often not presented as a single complete question but are distributed across multiple rounds of dialogue.

To ensure comparability of topic classification results across different platforms, this study provides supplementary annotation guidelines tailored to the consultation context of structured medical platforms, without altering the original topic definitions. The detailed supplementary rules are as follows.

## Autism symptom

**Definition:** Refers to posts in which patients or family members describe specific behavioral manifestations, developmental abnormalities, or daily responses of a child or individual, and consult physicians about whether these behaviors are abnormal, whether they are consistent with autism-related characteristics, or what the typical symptoms of autism are. The core focus of this category is whether the behaviors or manifestations themselves are abnormal, rather than issues related to formal diagnostic procedures or examination results. If the text explicitly asks “Is this autism?” or “How can autism be diagnosed?”, even when multiple symptoms are described, it should not be classified under this category but instead under “Autism Diagnosis.” On structured medical consultation platforms, this category should be used only when the text contains solely descriptive or inquiry-based consultation about behaviors or developmental manifestations (e.g., whether a symptom is severe or what the symptoms of autism are), does not include judgment-oriented questions such as “Is it autism?”, “diagnosis,” “confirmed,” “judgment,” or “which type,” and does not mention any examinations, scales, or assessment results.

**Keywords:** descriptions such as “doesn’t speak,” “avoids eye contact,” without explicit diagnostic requests; “what are the symptoms,” etc.

**Example posts:**

1. “My 6-month-old baby seems to show signs of autism. Is this flapping hands?”
2. “I would like to ask what symptoms babies with autism usually have?”

## Autism examination

**Definition:** Refers to posts in which patients or family members raise questions regarding autism-related examination items, assessment tools, or scale results, including whether a specific test is necessary, how to interpret examination or scale results, providing videos or images for the physician’s review, and asking about the role of examinations in the diagnostic process. If examination results are mentioned only as supplementary information and the core question concerns whether the condition is autism, the post should be classified under “Autism diagnosis.” The focus of this category is on the examination or assessment tool itself, including tests that have been completed or are planned. Even if the ultimate goal is diagnosis, the textual emphasis must be on the testing method or interpretation of results. The “Autism examination” category should be used only when the core question concerns how to understand, whether to conduct, or whether to trust a specific examination, scale, video, or result, and the post does not directly request a determination of whether it is autism. The emphasis is on the examination itself rather than diagnostic judgment or resource recommendation.

**Keywords:** “examination,” “scale,” “ADOS,” “intelligence test,” “assessment form,” “test results,” “whether to do a test,” etc.

**Example posts:**

1. “Could you please take a look at these videos and see if there is any problem?”
2. “Can genetic testing detect hereditary autism?”

## Autism diagnosis

**Definition:** Refers to posts in which patients or family members, based on described symptoms, explicitly focus on determining whether the condition is autism. This includes inquiries about autism subtypes, severity levels, diagnostic likelihood, evaluation conclusions, how to determine whether a child has autism, diagnostic procedures, or confirmation pathways, with the primary emphasis on autism diagnosis itself. When a post includes both symptom descriptions and examination results but centers on the question of “whether it is autism” or the “diagnostic pathway,” it should be classified under this category. This also includes posts that provide detailed accounts of a child’s behavioral manifestations or developmental history in order to seek a preliminary judgment or advice on diagnostic pathways, or that present examination results, scales, videos, or test findings as supporting evidence while asking whether they indicate autism. A post must be classified under “Autism diagnosis” if any of the following conditions are present: explicit questions such as “Is it autism?”, “Does it count as autism?”, “Is it confirmed?”, or “Can it be evaluated?”; inquiries about diagnostic procedures, diagnostic criteria, severity, or subtypes; or use of examinations, scales, or videos as evidence for the purpose of determining whether it is autism.

**Keywords:** “Is it autism?”, “please help judge,” “diagnosis,” “suspect,” “let’s discuss,” “I’m afraid it might be autism,” “what should I do,” etc.

**Example posts:**

1. “Please look at this test result. Does it count as autism now?”
2. “How is autism diagnosed in children?”

## Etiology and trigger-related inquiry

**Definition:** Refers to posts in which users raise questions regarding the causes, triggering mechanisms, or attribution of responsibility for autism, including genetic factors, prenatal or perinatal factors, parenting styles, environmental exposures, and related issues. The core focus of this category is the question “Why did this happen?”—that is, exploring the causes or potential contributing factors of autism. All inquiries specifically concerning the etiology or triggering factors of autism should be classified under this category.

**Keywords:** “genetic,” “did I cause this,” “during pregnancy,” “premature birth,” “vaccine,” “second child,” “family history,” etc.

**Example posts:**

1. “What are high-risk factors?”
2. “Is autism caused during pregnancy, or does it develop after birth?”

## Cost and financial burden inquiry

**Definition:** Refers to posts in which users raise questions about the economic costs and affordability of autism-related diagnosis, treatment, rehabilitation, or long-term interventions. The core focus of this category is financial cost, funding, or economic burden itself, rather than comparisons of specific institutions (e.g., “Which institution is better?”). All inquiries regarding expenses associated with autism-related treatment, intervention, or examinations should be classified under this category.

**Keywords:** “cost,” “expensive or not,” “how much was spent,” “reimbursement,” “can we afford it,” etc.

**Example posts:**

1. “How much does rehabilitation training usually cost?”
2. “How are the fees calculated?”

## Autism intervention

**Definition:** Refers to posts in which users seek advice regarding autism intervention approaches, treatment pathways, training methods, implementation strategies, and expected prognostic outcomes following intervention. This includes professional training interventions as well as implementation strategies in home or daily life contexts. The category covers intervention choices under different subtypes or severity levels and comparisons of the suitability and effectiveness of different intervention approaches. Such posts reflect concern about intervention outcomes and future development and essentially relate to decision-making regarding intervention and treatment. This includes inquiries about specific intervention methods, training strategies, whether improvement or recovery is possible, and future prognosis. However, it does not include general questions about the impact or severity of autism without reference to specific intervention methods, such as “Is autism serious?” or “Will autism affect the future?”

**Keywords:** “ABA,” “sensory integration training,” “speech therapy,” “family-based intervention,” “training intensity,” “intervention,” “don’t know what to do,” “rehabilitation methods,” etc.

**Example posts:**

1. “Do you have any advice for autism? Should we focus on diet, play more, or how should we approach rehabilitation?”
2. “The hospital hasn’t scheduled intervention courses for us yet. What should we mainly do for home-based intervention in the meantime?”

## Autism resource recommendation (evaluation)

**Definition:** Users seek recommendations, comparisons, or evaluations of autism-related diagnostic, treatment, or rehabilitation resources. These resources may include medical institutions (e.g., hospitals or departments), specific physicians (including questions about which department to register with), rehabilitation institutions, or practitioners (e.g., rehabilitation teachers, speech therapists, or behavior analysts). The core intent is to choose appropriate healthcare resources to reduce the risk of misdiagnosis and to lower decision uncertainty by drawing on others’ experiences. Posts that recommend or evaluate institutions, hospitals, or physicians—such as where to get examined, which department to visit, or where diagnosis/treatment is better—especially those emphasizing location, should be classified as Autism resource recommendation (evaluation).

**Keywords:** institution, hospital, doctor, teacher, recommendation, rehab center, reliable, scam/pitfall, cost/fees.

**Example posts:**

1. “Are there any rehabilitation centers in Anqing?”

2. “Not sure how effective the rehabilitation services are at the Hedong Hospital.”

## Other help-seeking

**Definition:** Refers to posts that contain a clear help-seeking intent but do not meet the criteria of any specific secondary subcategory; involve mixed issues such as autism combined with other medical conditions; contain multiple intertwined questions that are difficult to further disentangle; or address topics not directly related to autism. Posts with highly generalized or unclear problem descriptions, where the poster’s specific needs are difficult to identify, may also be included in this category. This category serves as a residual (catch-all) category and should be used only when classification into other specific subcategories is not possible. In particular, inquiries concerning examinations, diagnoses, or treatments unrelated to autism should be classified under “Other help-seeking.” For example, a question such as “I have an appointment on the 14th for a follow-up 4D ultrasound. Can a 4D ultrasound assess the condition of the heart?”—which is unrelated to autism—should be included in this category.

**Example posts:**

1. “Can this be played?”
2. “Is Simotang oral liquid the one for adults?”
