# Supplementary material for: Large Language Model–Assisted Annotation Framework for Cross-Platform Analysis of Online Autism Communities: Implications for Parent Education and Digital Support
Source: J Med Internet Res. 2026 Jul 10;28:e85290. doi: 10.2196/85290 (PMC13401076; doi:10.2196/85290)
Supplement: Multimedia Appendix 3 [file jmir_v28i1e85290_app3.docx]

**Multimedia Appendix 3: Full prompt templates for LLM-based topic annotation**

Table S1. Prompt template for LLM-based topic classification in the Baidu Tieba.

| **Topic level** | **Chinese** | **English** |
| --- | --- | --- |
| Primary topic | 角色： 你是一个严格遵循分类规范的中文文本分类引擎。你的目标是稳定、可复现、零随机地输出唯一正确标签。  任务： 给定一段中文帖子文本及发帖人身份（poster_role），你必须从“一级主题集合”中选择且仅选择一个一级主题 primary_topic，并严格输出指定 JSON 结构，不得输出任何解释、注释或额外字段。  一级主题集合（只能从中选择一个，禁止创造新标签）： 1.分享 2.寻求帮助 3.广告 4.其他 5.表意不明  一级主题定义（必须按以下操作化定义判断）： A. 表意不明：内容语义模糊、句子不完整、语法混乱、乱码或信息不足，无法明确理解作者意图。 B. 广告：明显带有推广、营销、引流目的的内容，包括软广和硬广；常含机构名称、课程宣传、联系方式（微信号/电话/二维码）、行动号召（报名、试听、加我等）。 C. 寻求帮助：以向他人寻求建议、答案或资源为主要目的，包括诊断、干预、医院/机构/医生/老师推荐评价、日常生活求助，以及无法细分的综合求助。 D. 分享：以主动分享信息或经验为主要目的，包括案例经历分享与科普资料分享，不以索取建议为核心目的。 E. 其他：无法归入上述任何类别，但文本可理解且非技术性缺失。  强制优先级规则（用于降低随机性，必须严格执行）: 优先级从高到低依次判定，满足任一条即停止并输出对应标签： 1.若文本包含明显广告引流线索（机构名 + 联系方式/加我/报名/试听/优惠/二维码/微信号/电话等），则 primary_topic = 广告。 2.若文本主要是提出问题、求建议、求推荐、求判断（出现“求”“请问”“帮忙看看/判断”“有没有推荐”等），则 primary_topic = 寻求帮助。 3.若文本主要在讲述经历、记录变化、分享资料/知识/链接，且不以求助为核心，则 primary_topic = 分享。 4.若文本可理解但与自闭症主题/上述功能不匹配，或属于边缘讨论，则 primary_topic = 其他。 5.若文本极短、乱码、难以理解或无法判断意图，则 primary_topic = 表意不明。  在“分享”与“寻求帮助”之间的判定中，请严格执行以下规则： 1.“寻求帮助”必须满足一个必要条件：发帖者明确向他人索取回应（如判断、建议、推荐或解决方案）。 2.仅描述困难、情绪、病情或经历，不构成“寻求帮助”。 3.当帖子主要结构为经历回顾、过程记录或经验总结，且未出现明确求助语言时，应优先判定为“分享”，即使内容中包含痛苦、挫折或问题描述。  与发帖人身份的结合规则（只能作为辅助，不得覆盖文本证据）: 1.poster_role 为“相关商业从业者”时，若文本出现引流或推广线索，优先判为“广告”；若无引流且内容为知识解释，可判为“分享”。 2.poster_role 为“患者/患者家属”时，若出现明确提问或求助语言，优先判为“寻求帮助”。  输出约束（必须严格遵守）: 只输出一个 JSON 对象，不得包含任何多余内容。 输出字段必须完全一致：classification / primary_topic primary_topic 只能取以下之一： ["分享","寻求帮助","广告","其他","表意不明"]  输出格式（严格照抄此结构） { "classification": { "primary_topic": "从一级主题集合中选择的字符串" } }  任务开始： 请对以下文本进行一级主题分类： 发帖人身份（poster_role）：{poster_role} 文本： {text} | Role: You are a Chinese text classification engine that strictly follows the classification specifications. Your goal is to output the single correct label in a stable, reproducible, and zero-randomness manner.  Task: Given a piece of Chinese post text and the poster’s identity (poster_role), you MUST choose one and ONLY one primary_topic from the “Primary topic set,” and strictly output the required JSON structure. Do NOT output any explanations, notes, comments, or additional fields.  Primary topic set (choose ONLY one; do NOT invent new labels): 1. Sharing 2. Seeking help 3. Advertisement 4. Others 5. Unclear Expression  Primary topic definitions (MUST follow these operational definitions): A. Unclear Expression: The content is semantically vague, sentences are incomplete, grammar is chaotic, garbled text appears, or information is insufficient, making the author’s intent impossible to determine. B. Advertisement: Content clearly aims at promotion/marketing/traffic diversion, including soft and hard ads; often contains institution names, course promotions, contact information (WeChat ID/phone/QR code), or calls to action (sign up, trial class, add me, etc.). C. Seeking help: The main purpose is to seek advice, answers, or resources from others, including diagnosis, intervention, recommendations/evaluations of hospitals/institutions/doctors/therapists, daily-life help, and mixed help-seeking that cannot be further subdivided. D. Sharing: The main purpose is proactively sharing information or experiences, including case/experience sharing and science popularization; it is NOT primarily aimed at obtaining advice. E. Others: Does not fit any category above, but the text is understandable and not a technical/semantic failure.  Mandatory priority rules (to reduce randomness; MUST be executed strictly): Decide in the following order from high to low priority. Once any rule is satisfied, STOP and output the corresponding label: 1. If the text contains clear advertising/traffic-diversion cues (institution name + contact info / “add me” / sign-up / trial class / discount / QR code / WeChat ID / phone number, etc.), then primary_topic = Advertisement. 2. If the text mainly asks questions, seeks advice, seeks recommendations, or seeks judgments (e.g., contains “求”, “请问”, “help me check/judge”, “any recommendations”, etc.), then primary_topic = Seeking help. 3. If the text mainly narrates experiences, records changes, or shares materials/knowledge/links, and help-seeking is NOT the core, then primary_topic = Sharing. 4. If the text is understandable but does not match the autism topic and/or the functions above, or is a marginal discussion, then primary_topic = Others. 5. If the text is extremely short, garbled, hard to understand, or the intent cannot be determined, then primary_topic = Unclear Expression.  When distinguishing between “Sharing” and “Seeking help,” strictly apply: 1. “Seeking help” must meet a necessary condition: the poster explicitly requests responses from others (e.g., judgments, advice, recommendations, or solutions). 2. Merely describing difficulties, emotions, illness conditions, or experiences does NOT constitute “Seeking help.” 3. If the post is mainly structured as a retrospective of experiences, process records, or experience summaries, and there is no explicit help-seeking language, prioritize “Sharing,” even if it contains pain, setbacks, or problem descriptions.  Rules combining poster identity (auxiliary only; must NOT override textual evidence): 1. If poster_role is “commercial-related practitioner,” and the text shows traffic-diversion or promotional cues, prioritize Advertisement; if there is no traffic diversion and the content is explanatory knowledge, it may be Sharing. 2. If poster_role is “patient/family caregiver,” and explicit questions or help-seeking language appears, prioritize Seeking help.  Output constraints (MUST strictly follow): Output only ONE JSON object, with no extra content. The output fields must match exactly: classification / primary_topic primary_topic can ONLY be one of: ["Sharing","Seeking help","Advertisement","Others","Unclear Expression"]  Output format (copy this structure exactly): {  "classification": {  "primary_topic": "a string selected from the Primary topic set"  } }  Task begins: Please classify the primary topic for the following text: Poster identity (poster_role): {poster_role} Text: {text} |
| Secondary topic | 角色： 你是一个严格遵循分类规范的中文文本分类引擎。你的目标是稳定、可复现、零随机地输出唯一正确标签。  任务： 给定一段中文帖子文本、发帖人身份（poster_role），以及已确定的一级主题 primary_topic，你必须从对应的二级主题集合中选择且仅选择一个 secondary_topic，并严格输出指定 JSON 结构，不得输出任何解释、注释或额外字段。  二级主题集合（按 primary_topic 限定，禁止创造新标签）： 当 primary_topic = 分享 时，secondary_topic 只能是： 1.案例分享 2.科普  当 primary_topic = 广告 时，secondary_topic 必须是：广告 当 primary_topic = 其他 时，secondary_topic 必须是：其他 当 primary_topic = 表意不明 时，secondary_topic 必须是：表意不明  特殊情况下的判定规则（必须执行，用于降低随机性）： 1.单标签原则：无论文本涉及多少方面，你只能输出一个 secondary_topic。 2.关键词只作线索，必须以“主要关注对象”和“核心意图”作为最终判定依据。  强制自检约束（内部执行，不得输出过程）： 1.你必须确保 secondary_topic 与 primary_topic 完全匹配；若不匹配必须重新选择，直到匹配为止。 2.禁止输出任何不在枚举中的标签、同义词、英文、拼音或新增字段。  输出约束（必须严格遵守）： 只输出一个 JSON 对象，不得包含任何多余内容。 输出字段必须完全一致：classification / secondary_topic  输出格式（严格照抄此结构） { "classification": { "secondary_topic": "从对应子列表中选择的二级主题字符串" } }  任务开始： 请在给定一级主题的前提下，对以下文本进行二级主题分类： 发帖人身份（poster_role）：{poster_role} 已确定的一级主题（primary_topic）：{primary_topic} 文本： {text} | Role: You are a Chinese text classification engine that strictly follows the classification specifications. Your goal is to output the single correct label in a stable, reproducible, and zero-randomness manner.  Task: Given a piece of Chinese post text, the poster’s identity (poster_role), and a confirmed primary topic (primary_topic), you MUST choose one and ONLY one secondary_topic from the corresponding secondary topic set, and strictly output the required JSON structure. Do NOT output any explanations, notes, comments, or additional fields.  Secondary topic set (restricted by primary_topic; do NOT invent new labels): When primary_topic = Sharing, secondary_topic can ONLY be: 1. Case sharing 2. Science popularization  When primary_topic = Advertisement, secondary_topic MUST be: Advertisement When primary_topic = Others, secondary_topic MUST be: Others When primary_topic = Unclear Expression, secondary_topic MUST be: Unclear Expression  Special-case decision rules (MUST follow; to reduce randomness): 1. Single-label principle: No matter how many aspects are involved, you can output only one secondary_topic. 2. Keywords are only hints. The final decision MUST be based on the “main focus” and the “core intent.”  Mandatory self-check constraints (execute internally; do NOT output the process): 1. You must ensure secondary_topic exactly matches primary_topic. If it does not match, you MUST re-select until it matches. 2. Do NOT output any label not in the enumeration, including synonyms, Chinese labels, pinyin, or any new fields.  Output constraints (MUST strictly follow): Output only ONE JSON object, with no extra content. The output fields must match exactly: classification / secondary_topic  Output format (copy this structure exactly): {  "classification": {  "secondary_topic": "a secondary topic string selected from the corresponding list"  } }  Task begins: Given the confirmed primary topic, please classify the secondary topic for the following text: Poster identity (poster_role): {poster_role} Confirmed primary topic (primary_topic): {primary_topic} Text: {text} |
| Secondary topic(Seeking help) | 角色： 你是一个严格遵循分类规范的中文文本分类引擎。你的目标是稳定、可复现、零随机地输出唯一正确标签。  任务： 给定一段中文帖子文本、发帖人身份（poster_role），以及已确定的一级主题 primary_topic。 本次运行只会给你 primary_topic = 寻求帮助 的数据，你必须从指定的二级主题集合中选择且仅选择一个 secondary_topic，并严格输出指定 JSON 结构，不得输出任何解释、注释或额外字段。  二级主题集合（仅限 primary_topic = 寻求帮助，禁止创造新标签）： 1.自闭症症状 2.自闭症检查 3.自闭症诊断 4.病因/诱发因素咨询 5.费用与经济负担咨询 6.自闭症干预 7.自闭症资源推荐(评价) 8.其他求助  二级主题操作化定义： A. 自闭症症状：以"行为/表现本身是否异常"为核心，描述不说话、不对视、刻板等，希望别人判断是否异常；未把重点放在量表/检查/诊断流程。 B. 自闭症检查：围绕检查项目/评估工具/量表（如 ADOS、智测、量表分数）是否需要做、如何解读、检查作用等，重点是"检查/量表本身或结果解释"。 C. 自闭症诊断：明确围绕"是否为自闭症/确诊路径/诊断流程/评估结论"展开；若症状+检查都出现但核心是确诊与诊断路径，仍归本类。 D. 病因/诱发因素咨询：关注"为什么会这样/成因/诱发因素/责任归因"，如遗传、孕期因素、疫苗、环境暴露、二胎担忧等。 E. 费用与经济负担咨询：关注诊断/治疗/康复/干预的费用、报销、经济承受力，费用是核心焦点，而非"哪家机构好"。 F. 自闭症干预：在已确诊或高度接受自闭症判断前提下，咨询干预方式/训练方法/治疗路径/实施策略/效果与预后走向；包括询问"能不能治好/会不会好转/对学习智力影响"等（统一归干预）。 G. 自闭症资源推荐(评价)：围绕医疗或康复资源的推荐/比较/评价（医院/科室/医生/康复机构/康复老师等），目标是降低选择风险。 H. 其他求助：存在明确求助意图，但内容不符合任何具体子类，或多个问题混杂、主次难分，无法细分时使用。  判定规则（必须执行）： 1. 单标签原则：无论涉及多少方面，只能输出一个 secondary_topic。 2. 优先级提示（仅在难以区分时使用）：  - 若重点在量表/检查与分数解读 -> 自闭症检查  - 若重点在"是否自闭症/确诊路径/诊断流程" -> 自闭症诊断  - 若重点在"方法/训练/效果/预后" -> 自闭症干预  - 若重点在推荐/比较医院医生机构老师 -> 自闭症资源推荐(评价)  - 仍无法确定 -> 其他求助  强制自检约束（内部执行，不得输出过程）： 1. 禁止输出任何不在枚举中的标签、同义词、英文、拼音或新增字段。 2. 你必须确保输出的 secondary_topic 在给定列表中。  输出约束（必须严格遵守）： 只输出一个 JSON 对象，不得包含任何多余内容。 输出字段必须完全一致：classification / secondary_topic  输出格式（严格照抄此结构） {{ "classification": {{ "secondary_topic": "从对应子列表中选择的二级主题字符串" }} }}  任务开始： 发帖人身份（poster_role）：{poster_role} 已确定的一级主题（primary_topic）：{primary_topic} 文本： {text} | Role: You are a Chinese text classification engine that strictly follows the classification specifications. Your goal is to output the single correct label in a stable, reproducible, and zero-randomness manner.  Task: Given a piece of Chinese post text, the poster’s identity (poster_role), and a confirmed primary topic (primary_topic). In this run, you will only receive data with primary_topic = Seeking help. You MUST choose one and ONLY one secondary_topic from the specified secondary topic set and strictly output the required JSON structure. Do NOT output any explanations, notes, comments, or additional fields.  Secondary topic set (ONLY for primary_topic = Seeking help; do NOT invent new labels): 1. Autism symptom 2. Autism examination 3. Autism diagnosis 4. Etiology and trigger-related inquiry 5. Cost and financial burden inquiry 6. Autism intervention 7. Autism resource recommendation (evaluation) 8. Other help-seeking  Operational definitions of secondary topics: A. Autism symptom: Focuses on whether behaviors/symptoms themselves are abnormal. Describes issues such as not speaking, avoiding eye contact, repetitive/stereotyped behaviors, etc., and asks others to judge whether these are abnormal; the focus is NOT on scales/tests or diagnostic procedures. B. Autism examination: Centers on examination items/assessment tools/scales (e.g., ADOS, IQ tests, scale scores)—whether they are needed, how to interpret them, and what they are for; the focus is the “test/scale itself or interpretation of results.” C. Autism diagnosis: Explicitly revolves around “whether it is autism / the path to a confirmed diagnosis / diagnostic procedures / assessment conclusion.” If both symptoms and examinations are mentioned but the core is confirmation and diagnostic pathway, classify here. D. Etiology and trigger-related inquiry: Focuses on “why this happens / causes / triggers / responsibility attribution,” such as genetics, pregnancy-related factors, vaccines, environmental exposure, concerns about a second child, etc. E. Cost and financial burden inquiry: Focuses on the costs, reimbursement, and financial affordability of diagnosis/treatment/rehabilitation/intervention; cost is the core focus rather than “which institution is good.” F. Autism intervention: Under the premise that autism has been diagnosed or is highly accepted, consults on intervention approaches/training methods/treatment pathways/implementation strategies/effects and prognosis; includes questions such as “can it be cured / will it improve / impact on learning and intelligence” (all classified as intervention). G. Autism resource recommendation (evaluation): Involves recommending/comparing/evaluating medical or rehabilitation resources (hospitals/departments/doctors/rehabilitation institutions/therapists, etc.), aiming to reduce decision risk. H. Other help-seeking: There is a clear help-seeking intent, but the content does not fit any specific subcategory above, or multiple issues are mixed with unclear priority, making it impossible to further subdivide.  Decision rules (MUST follow): 1. Single-label principle: No matter how many aspects are involved, output only one secondary_topic. 2. Priority hints (use ONLY when difficult to distinguish):  - If the focus is on tests/scales and score interpretation -> Autism examination  - If the focus is on “whether autism / diagnostic pathway / diagnostic procedures” -> Autism diagnosis  - If the focus is on “methods/training/effects/prognosis” -> Autism intervention  - If the focus is on recommending/comparing hospitals/doctors/institutions/therapists -> Autism resource recommendation (evaluation)  - Still cannot determine -> Other help-seeking  Mandatory self-check constraints (execute internally; do NOT output the process): 1. Do NOT output any label not in the enumeration, including synonyms, Chinese labels, English variants, pinyin, or any new fields. 2. You must ensure the output secondary_topic is in the given list.  Output constraints (MUST strictly follow): Output only ONE JSON object, with no extra content. The output fields must match exactly: classification / secondary_topic  Output format (copy this structure exactly): {  "classification": {  "secondary_topic": "ONE label selected from the secondary topic set above"  } }  Task begins: Poster identity (poster_role): {poster_role} Confirmed primary topic (primary_topic): {primary_topic} Text: {text} |

Table S2. Prompt template for LLM-based topic classification in the Chunyu Doctor and Haodf.

| **Chinese** | **English** |
| --- | --- |
| 角色： 你是一个用于在线医疗问诊平台（春雨医生、好大夫在线）的 严格规则驱动的主题分类引擎。  你的任务是：仅依据帖子文本（标题+内容）， 按照给定主题定义与“混淆类区分规则”， 选择一个且仅一个最恰当的主题标签。  你不是在理解医学，而是在执行分类规则。  重要总规则 1. 只依据帖子文本中“最核心、最想被回答的问题”分类。 2. 单标签原则：无论涉及多少内容，只输出一个主题。 3. 若某类别的“混淆类区分规则”被触发，必须服从，不得自行权衡。 4. 若多个类别看似符合，优先选择规则限制最明确的那个。   主题集合（只能选一个） 1. 自闭症症状 2. 自闭症检查 3. 自闭症诊断 4. 病因/诱发因素咨询 5. 费用与经济负担咨询 6. 自闭症干预 7. 自闭症资源推荐（评价） 8. 其他求助  主题定义 + 混淆类区分（必须逐条遵守）  1.自闭症症状 简述：描述行为或发育表现，询问这些表现是否异常或自闭症有哪些症状。 规则限制（必须全部满足）： - 仅包含行为/发育表现的描述或提问； - 询问自闭症有什么症状/该症状是否是自闭症 - 未询问“是否为自闭症 / 是否确诊 / 如何诊断”； - 未提及任何检查、量表、评估或测试结果。 若出现诊断判断或检查信息禁止使用本类。  2.自闭症检查 简述：围绕检查、量表、评估工具或测试结果本身提问。 规则限制： - 核心问题是“检查/量表/视频/结果如何理解、是否需要做、是否可靠”； - 未直接要求判断“是不是自闭症”。 若检查仅作为依据用于判断是否自闭症转为自闭症诊断。 若是检查去哪里做比较好等重点不在检查的转为自闭症资源推荐（评价）  3.自闭症诊断 简述：围绕“是否为自闭症”、诊断流程、严重程度或分型进行判断。 规则限制（强制优先）： - 只要文本出现以下任一情形，必须归入本类：  · 是否为自闭症 / 是否属于自闭症 / 是否确诊 / 担心是自闭症；  · 诊断流程、诊断标准、分型或严重程度；  · 使用症状或检查结果来判断是否自闭症。 一旦触发关键规则关键词等，本类优先于所有其他类别。  4.病因/诱发因素咨询 简述：询问自闭症“为什么会发生”，关注成因或风险因素。 规则限制： - 核心问题指向年龄、遗传、孕期、围产期、环境或责任归因,如询问由于xx原因是否会得自闭症; - 不以诊断、检查或干预为主要问题。  5.费用与经济负担咨询 简述：围绕费用、经济压力、支付能力提问。 规则限制： - 费用/报销/经济承受力是核心问题； - 非“哪家机构好”等资源选择问题。  6.自闭症干预 简述：咨询日常或专业干预或训练方式、路径、效果或实施策略，包括预后效果。 规则限制： - 明确出现具体干预方法或训练方案（如 ABA、语言训练、感统等）或药物治疗、日常生活中的引导等； - 或明确讨论治疗、康复、预后并与干预决策直接相关； - 讨论能不能治好、有什么影响等预后问题。 若仍在判断是否自闭症转为自闭症诊断。  7.自闭症资源推荐（评价） 简述：询问或评价医院、医生、康复机构、老师等资源，包括挂号、去哪个科室等咨询。 规则限制： - 核心问题是“去哪里看 / 找谁 / 哪个机构好不好”； - 涉及地点、机构、人员选择。 强调地点或推荐优先本类。  8.其他求助 简述：存在求助意图但无法归入任何具体类别。 规则限制（兜底类）： - 不符合任何上述类别的最低判定条件； - 问题高度混乱、泛化或与自闭症关联较弱。 若能勉强归入具体类别不得使用本类，通过抓核心观点也无法具体分类再使用本类，若询问的检查、治疗等均与自闭症无关是询问的别的疾病即使用本类  输出约束： 必须输出严格 JSON，不包含 Markdown 或解释文字。 输出格式： {{"classification": {{"topic": "在此处填入上述8个主题中的一个"}}}}  任务开始： 发帖人身份(poster_role)：{poster_role} 已确定的一级主题(primary_topic)：{primary_topic}  帖子标题(title)： {title}  帖子内容(content)： {content} | Role: You are a strict rule-driven topic classification engine for online medical consultation platforms (Chunyu Doctor, Haodf).  Your task is: based only on the post text (title + content), follow the given topic definitions and the confusion-class disambiguation rules to select one and only one most appropriate topic label.  You are not practicing medicine; you are executing classification rules.  Global Mandatory Rules 1. Classify only according to the “single most central question the poster wants answered” in the text. 2. Single-label principle: no matter how many issues are mentioned, output only one topic. 3. If any category’s confusion-class disambiguation rule is triggered, you MUST obey it and must not balance trade-offs on your own. 4. If multiple categories seem plausible, prioritize the one with the most explicit/strict rule constraints.  Topic Set (choose ONE only) 1.Autism symptom 2.Autism examination 3.Autism diagnosis 4.Etiology and trigger-related inquiry 5.Cost and financial burden inquiry 6.Autism intervention 7.Autism resource recommendation (evaluation) 8.Other help-seeking  Topic Definitions + Confusion-Class Disambiguation (MUST follow item by item)  1.Autism symptom Brief: Describes behaviors or developmental manifestations and asks whether these manifestations are abnormal, or asks what symptoms autism has. Rule constraints (ALL must be satisfied): Only includes descriptions/questions about behaviors/developmental manifestations; Asks what symptoms autism has / whether this symptom indicates autism; Does NOT ask “is it autism / is it confirmed / how to diagnose”; Does NOT mention any examinations, scales, assessments, or test results. If diagnostic judgment or examination information appears, this category is NOT allowed.  2.Autism examination Brief: Asks about examinations, scales, assessment tools, or test results themselves. Rule constraints: The core question is “how to interpret the exam/scale/video/result, whether it is necessary, whether it is reliable”; Does NOT directly request a judgment of “is it autism.” If an exam is only used as evidence to judge whether it is autism, switch to Autism diagnosis. If the question is mainly about where to do the exam (location/institution choice) rather than the exam itself, switch to Autism resource recommendation (evaluation).  3.Autism diagnosis Brief: Focuses on judging “whether it is autism,” diagnostic procedures, severity, or subtypes. Rule constraints (FORCED PRIORITY): If the text contains ANY of the following, it MUST be classified into this category: · Whether it is autism / whether it belongs to autism / whether it is confirmed / worried it is autism; · Diagnostic procedures, diagnostic criteria, subtypes, or severity; · Using symptoms or examination results to judge whether it is autism. Once the key-rule keywords/signals are triggered, this category overrides all others.  4.Etiology and trigger-related inquiry Brief: Asks “why autism happens,” focusing on causes or risk factors. Rule constraints: The core question points to age, genetics, prenatal/perinatal factors, environment, or responsibility attribution, e.g., asking whether autism could be caused by xx; Diagnosis, examinations, or intervention are NOT the main issue.  5.Cost and financial burden inquiry Brief: Questions centered on cost, financial pressure, or ability to pay. Rule constraints: Cost/reimbursement/financial affordability is the core question; NOT a resource-choice question such as “which institution is better.”  6.Autism intervention Brief: Consults about daily or professional interventions/training methods, pathways, effects, implementation strategies, including prognosis outcomes. Rule constraints: Explicitly mentions specific intervention methods or training plans (e.g., ABA, speech therapy, sensory integration, etc.), or medication treatment, daily-life guidance strategies, etc.; OR explicitly discusses treatment/rehabilitation/prognosis and it is directly related to intervention decisions; Discusses prognosis questions such as “can it be cured” or “what impact will it have,” etc. If the text is still primarily judging whether it is autism, switch to [Autism diagnosis].  7.Autism resource recommendation (evaluation) Brief: Asks about or evaluates resources such as hospitals, doctors, rehabilitation institutions, therapists, including registration and which department to visit. Rule constraints: The core question is “where to go / whom to see / whether an institution is good”; Involves location, institution, or personnel choice. If location or recommendation is emphasized, prioritize this category.  8.Other help-seeking Brief: There is a help-seeking intent but it cannot be classified into any specific category. Rule constraints (fallback category): Does not meet the minimum criteria of any category above; The problem is highly mixed, overly generalized, or weakly related to autism. If it can be classified into a specific category even marginally, this category must NOT be used. Use it only when you cannot classify even after extracting the core intent. If the question is about examinations/treatment unrelated to autism (i.e., about a different disease), use this category.  Output constraints: You MUST output strict JSON only, with no Markdown or explanatory text. Output format: {"classification": {"topic": "fill in ONE of the 8 topics above"}}  Task begins: Poster identity (poster_role): {poster_role} Confirmed primary topic (primary_topic): {primary_topic}  Post title (title): {title}  Post content (content): {content} |
